# Integrated epigenetic and genetic analysis identifies markers of prognostic significance in pediatric acute myeloid leukemia

**DOI:** 10.18632/oncotarget.25475

**Published:** 2018-06-01

**Authors:** Jatinder K. Lamba, Xueyuan Cao, Susana C. Raimondi, Roya Rafiee, James R. Downing, Shi Lei, Tanja Gruber, Raul C. Ribeiro, Jeffrey E. Rubnitz, Stanley B. Pounds

**Affiliations:** ^1^ Department of Pharmacotherapy and Translational Research, Center for Pharmacogenomics, University of Florida, Gainesville, FL, USA; ^2^ Department of Biostatistics, St. Jude Children's Research Hospital, Memphis, TN, USA; ^3^ Department of Pathology, St. Jude Children's Research Hospital, Memphis, TN, USA; ^4^ Department of Oncology, St. Jude Children's Research Hospital, Memphis, TN, USA

**Keywords:** AML, methylation, pediatrics, leukemia

## Abstract

Acute myeloid leukemia (AML) may be an epigenetically-driven malignancy because it harbors fewer genomic mutations than other cancers. In recent studies of AML in adults, DNA methylation patterns associate with clinical risk groups and prognosis. However, thorough evaluations of methylation in pediatric AML have not been done. Therefore, we performed an integrated analysis (IA) of the methylome and transcriptome with clinical outcome in 151 pediatric patients from the multi-center AML02 clinical trial discovery cohort. Intriguingly, reduced methylation and increased expression of DNMT3B was associated with worse clinical outcomes (IA *p* ≤ 10^−5^; *q* ≤ 0.002). In particular, greater DNMT3B expression associated with worse minimal residual disease (MRD; *p* < 10^−5^; *q* = 0.01), a greater rate of relapse or resistant disease (RR) (*p* = 0.00006; *q* = 0.06), and event-free survival (EFS; *p* = 0.00003; *q* = 0.04). Also, greater DNMT3B expression associated with greater genome-wide methylation burden (GWMB; *R* = 0.39; *p* = 10^−6^) and greater GWMB associated with worse clinical outcomes (IA *p* < 10^−5^). In an independent validation cohort of 132 similarly treated AAML0531 clinical trial patients, greater DNMT3B expression associated with greater GWMB, worse MRD, worse RR, and worse EFS (all *p* < 0.03); also, greater GWMB associated with worse MRD (*p* = 0.004) and EFS (*p* = 0.037). These results indicate that DNMT3B and GWMB may have a central role in the development and prognosis of pediatric AML.

## INTRODUCTION

Acute myeloid leukemia (AML) is a heterogeneous disease with genetic mutations and chromosomal abnormalities that define clinical risk-groups and treatment outcome. Although recurrent mutations are less common in AML than in other cancers, advances in recent years have shown that there is a significant deregulation of DNA methylation in adult AML [1, 2]. Aberrant DNA methylation is one of the most frequent epigenetic markers of leukemogenesis in adult AML [3–8]. Figueroa *et al.*, reported 16 differentially methylated regions of which 11 were associated with previously known cytogenetic or genetic alterations [9]. Several other reports have also shown differentially methylated profiles related with AML subtypes as well as methylation signatures predictive of clinical outcome [1, 3, 10–13]. Recurrent mutations of prognostic significance in epigenetic genes such as DNMT3A, TET2, ASXL1, CREBP/KAT3A and EZH2 have also been reported in adult AML [14, 15]. Clinical trials in adult AML are exploring ways to therapeutically target these epigenetic processes with hypomethylating agents such as azacytidine and decitabine [8]. In spite of all these advances in adult AML, the genome-wide methylation studies of childhood AML are lacking. Extrapolation of methylation landscape of adult AML might not be appropriate for pediatric AML given differences in AML biology within these groups [16], as well as the knowledge that age-related differences between the normal methylomes of children and adults exists [17]. Thus, there is an unmet need to establish and characterize the DNA methylome of pediatric AML. In this study, we report integrated evaluation of epigenetic-transcriptional features that associate with the clinical outcome in pediatric AML from multi-center AML02 trial. We further validated our results in the independent cohort of pediatric patients enrolled on the standard arm of COG-AAML0531 clinical trail from TARGET database.

## RESULTS

### Gene methylation and expression associate with clinical outcomes in AML02

**Figure 1:**
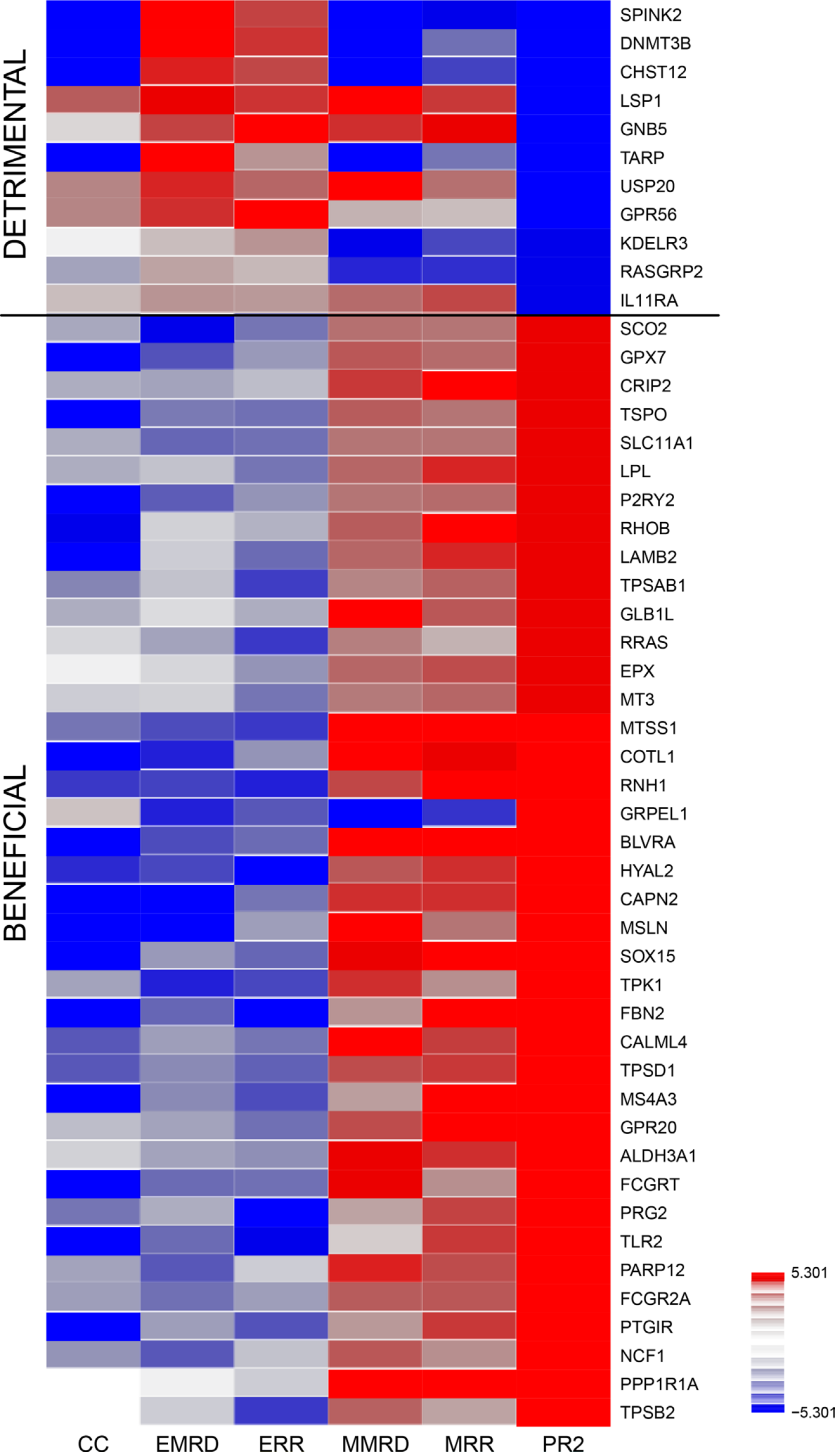
Therapeutically beneficial and detrimental patterns of association detected by the CC-PROMISE procedure Association Heatmap for top 50 genes by CC-PROMISE analysis. Each block of the heatmap illustrates the results of a statistical test of association. CC-PROMISE identified 11 genes for which increased expression associated with poorer prognosis (detrimental) and 39 for which increased expression associated with better prognosis (beneficial). The color indicates the direction of association (red = positive; blue = negative) and the intensity represents statistical significance on the log_10_ *p*-value scale. Each row provides association analysis results for one gene. The columns provide results for the associations of methylation with expression (ME), expression and MRD (EMRD), expression and RR (ERR), methylation and MRD (MMRD), methylation and MRD (MMRD) and the integrated CC-PROMISE result (CCPR). MRD: Minimal Residual Disease; RR: Risk of relapse or resistant disease.

We used the integrated data analysis method canonical correlation with projection on the most interesting statistical evidence (CC-PROMISE [18]) and identified 50 genes with correlated methylation and expression levels that were concordantly associated with clinical outcomes in the AML02 cohort (Table 1; Figure 1; *p* ≤ 0.00001; *q* ≤ 0.002). After adjusting for clinical risk group, 45 of these 50 genes remained significant at *p* ≤ 0.05 (Supplementary Figure 1; Supplementary Table 2), suggesting that these associations are not entirely attributable to differences across risk groups. Several of the identified 50 genes have known biological or clinical relevance for AML. Among these genes were: MTSS1 (Metastasis suppressor) is regulated by DNMT3B [19] and has been associated with better outcome in normal karyotype AML [20]; PRG2, a proteoglycan with role in myeloid differentiation, PARP12, involved in death-receptor and MAPK signaling, members of tryptase family (TPSD1, TPSB2, TPSAB1). LPL, CAPN2, FBN2, MS4A3, MT3, MSLN and Ras related genes (RHOB and RRAS), TIAF1 have been shown to have relevance in AML and other malignancies [21–31]. Genes identified as predictive of detrimental outcome included SPINK2, DNMT3B, CHST12, TARP, KDELR3, RASGRP2, LSP1, GNB5, USP20, GPR56 and IL11RA were associated with inferior outcome in terms of MRD22, RR and EFS as the endpoints) were predictive of poor outcome (Figure 1). GPR56 has been implicated in the development of AML in mice [21] and its expression has been associated with high risk AML and poor outcome [22].

**Table 1:** Top 50 genes identified by unstratified CC-PROMISE analysis to be significantly associated with MRD and RR at PROMISE *p* < 0.00001

Association Variables	Methylation-Expression Association by Canonical Correlation	Association of Expression with Clinical Outcomes					Association of Methylation with Clinical Outcomes				Integrated Association of Methylation and Expression with Clinical Outcomes	
		MRD		RR		MRD		RR				
**Gene**	***R***	***p***	***R***	***p***	***R***	***p***	***R***	***p***	***R***	***p***	***R***	***p***
MTSS1	−0.68	0.00157	−0.31	0.00019	−0.31	0.00007	0.41	0.00000	0.41	0.00000	0.359	0.00000
COTL1	−0.65	0.00000	−0.35	0.00002	−0.22	0.00628	0.52	0.00000	0.33	0.00001	0.355	0.00000
SPINK2	−0.79	0.00000	0.39	0.00000	0.31	0.00013	−0.36	0.00000	−0.35	0.00001	−0.352	0.00000
**DNMT3B**	**−0.74**	**0.00000**	**0.42**	**0.00000**	**0.32**	**0.00006**	**−0.40**	**0.00000**	**−0.26**	**0.00108**	**−0.352**	**0.00000**
CHST12	−0.65	0.00000	0.36	0.00002	0.30	0.00017	−0.42	0.00000	−0.31	0.00012	−0.345	0.00000
LSP1	0.79	0.00044	0.35	0.00001	0.32	0.00006	0.38	0.00000	0.33	0.00007	−0.344	0.00000
RNH1	−0.70	0.00007	−0.32	0.00013	−0.36	0.00002	0.32	0.00017	0.37	0.00000	0.342	0.00000
GNB5	0.60	0.15400	0.31	0.00014	0.36	0.00000	0.34	0.00004	0.35	0.00001	−0.341	0.00000
TARP	−0.71	0.00000	0.41	0.00000	0.22	0.00726	−0.46	0.00000	−0.26	0.00142	−0.338	0.00000
GRPEL1	0.45	0.05906	−0.37	0.00002	−0.29	0.00033	−0.37	0.00000	−0.32	0.00006	0.337	0.00000
BLVRA	−0.68	0.00001	−0.31	0.00020	−0.27	0.00088	0.40	0.00000	0.35	0.00000	0.331	0.00000
HYAL2	−0.62	0.00003	−0.31	0.00018	−0.37	0.00000	0.30	0.00032	0.32	0.00005	0.325	0.00000
CAPN2	−0.84	0.00000	−0.35	0.00000	−0.26	0.00145	0.34	0.00005	0.33	0.00004	0.321	0.00000
MSLN	−0.78	0.00000	−0.40	0.00000	−0.21	0.00981	0.38	0.00000	0.26	0.00145	0.313	0.00000
USP20	0.54	0.00320	0.33	0.00003	0.27	0.00069	0.39	0.00000	0.26	0.00110	−0.312	0.00000
SOX15	−0.63	0.00000	−0.23	0.00797	−0.27	0.00068	0.36	0.00001	0.37	0.00000	0.307	0.00000
TPK1	−0.55	0.01567	−0.35	0.00002	−0.30	0.00018	0.35	0.00005	0.23	0.00474	0.305	0.00000
FBN2	−0.69	0.00001	−0.29	0.00069	−0.35	0.00000	0.23	0.00753	0.33	0.00000	0.298	0.00000
CALML4	−0.44	0.00039	−0.21	0.01207	−0.26	0.00142	0.37	0.00000	0.31	0.00009	0.288	0.00000
TPSD1	−0.49	0.00037	−0.24	0.00381	−0.27	0.00052	0.30	0.00021	0.32	0.00008	0.284	0.00000
MS4A3	−0.57	0.00000	−0.24	0.00366	−0.30	0.00021	0.21	0.01173	0.38	0.00000	0.283	0.00000
GPR20	−0.47	0.04763	−0.21	0.01395	−0.26	0.00115	0.31	0.00023	0.35	0.00000	0.281	0.00000
ALDH3A1	−0.47	0.12037	−0.21	0.01336	−0.22	0.00570	0.36	0.00001	0.32	0.00005	0.277	0.00000
FCGRT	−0.65	0.00000	−0.28	0.00099	−0.25	0.00134	0.35	0.00001	0.22	0.00531	0.277	0.00000
PRG2	−0.42	0.00164	−0.19	0.02274	−0.39	0.00000	0.21	0.01313	0.30	0.00013	0.273	0.00000
GPR56	0.53	0.00292	0.35	0.00004	0.38	0.00000	0.19	0.02885	0.16	0.04329	−0.269	0.00000
TLR2	−0.60	0.00000	−0.28	0.00085	−0.34	0.00001	0.14	0.10899	0.32	0.00007	0.268	0.00000
PARP12	−0.40	0.01553	−0.30	0.00032	−0.13	0.10865	0.34	0.00002	0.30	0.00021	0.267	0.00000
FCGR2A	−0.33	0.01121	−0.27	0.00110	−0.21	0.01031	0.30	0.00040	0.29	0.00037	0.266	0.00000
PTGIR	−0.67	0.00000	−0.21	0.01130	−0.30	0.00028	0.22	0.00835	0.32	0.00008	0.263	0.00000
NCF1	−0.26	0.00601	−0.30	0.00037	−0.15	0.06345	0.30	0.00038	0.22	0.00565	0.243	0.00000
PPP1R1A	−0.21	0.79607	−0.05	0.55787	−0.14	0.08891	0.43	0.00000	0.35	0.00000	0.243	0.00000
TPSB2	−0.02	0.83668	−0.14	0.08947	−0.32	0.00008	0.29	0.00062	0.20	0.01259	0.239	0.00000
SCO2	−0.47	0.01700	−0.33	0.00001	−0.26	0.00172	0.27	0.00120	0.25	0.00157	0.278	0.00001
GPX7	−0.59	0.00000	−0.30	0.00029	−0.21	0.00858	0.30	0.00034	0.27	0.00098	0.271	0.00001
CRIP2	−0.50	0.02055	−0.21	0.01369	−0.16	0.05201	0.34	0.00008	0.38	0.00000	0.271	0.00001
KDELR3	−0.32	0.52818	0.16	0.05209	0.22	0.00680	−0.40	0.00001	−0.30	0.00017	−0.270	0.00001
TSPO	−0.66	0.00000	−0.26	0.00191	−0.26	0.00110	0.30	0.00044	0.26	0.00142	0.269	0.00001
SLC11A1	−0.59	0.02452	−0.29	0.00069	−0.26	0.00114	0.26	0.00173	0.26	0.00139	0.267	0.00001
RASGRP2	−0.60	0.01403	0.21	0.01415	0.17	0.03534	−0.35	0.00003	−0.32	0.00004	−0.261	0.00001
LPL	−0.45	0.02421	−0.16	0.05661	−0.26	0.00144	0.29	0.00073	0.33	0.00003	0.260	0.00001
P2RY2	−0.67	0.00000	−0.29	0.00050	−0.22	0.00646	0.27	0.00163	0.26	0.00090	0.260	0.00001
RHOB	−0.55	0.00001	−0.13	0.11690	−0.18	0.02597	0.29	0.00050	0.43	0.00000	0.257	0.00001
LAMB2	−0.63	0.00000	−0.14	0.10805	−0.26	0.00085	0.28	0.00074	0.33	0.00003	0.252	0.00001
IL11RA	0.31	0.04458	0.22	0.00756	0.21	0.00959	0.28	0.00096	0.29	0.00018	−0.251	0.00001
TPSAB1	−0.45	0.00338	−0.16	0.05424	−0.31	0.00009	0.25	0.00311	0.27	0.00063	0.250	0.00001
GLB1L	−0.43	0.02302	−0.11	0.20497	−0.19	0.02063	0.39	0.00000	0.29	0.00033	0.243	0.00001
RRAS	−0.33	0.16396	−0.21	0.01259	−0.31	0.00008	0.26	0.00231	0.18	0.02939	0.239	0.00001
EPX	−0.27	0.52806	−0.12	0.15835	−0.22	0.00630	0.28	0.00070	0.30	0.00020	0.231	0.00001
MT3	−0.35	0.09042	−0.13	0.12471	−0.25	0.00170	0.26	0.00175	0.27	0.00081	0.228	0.00001

Correlation between methylation and expression for each gene is shown in column title “Methylation/Expression correlation”

### DNMT3B methylation and expression are prognostic factors in the AML02 discovery cohort

**Figure 2:**
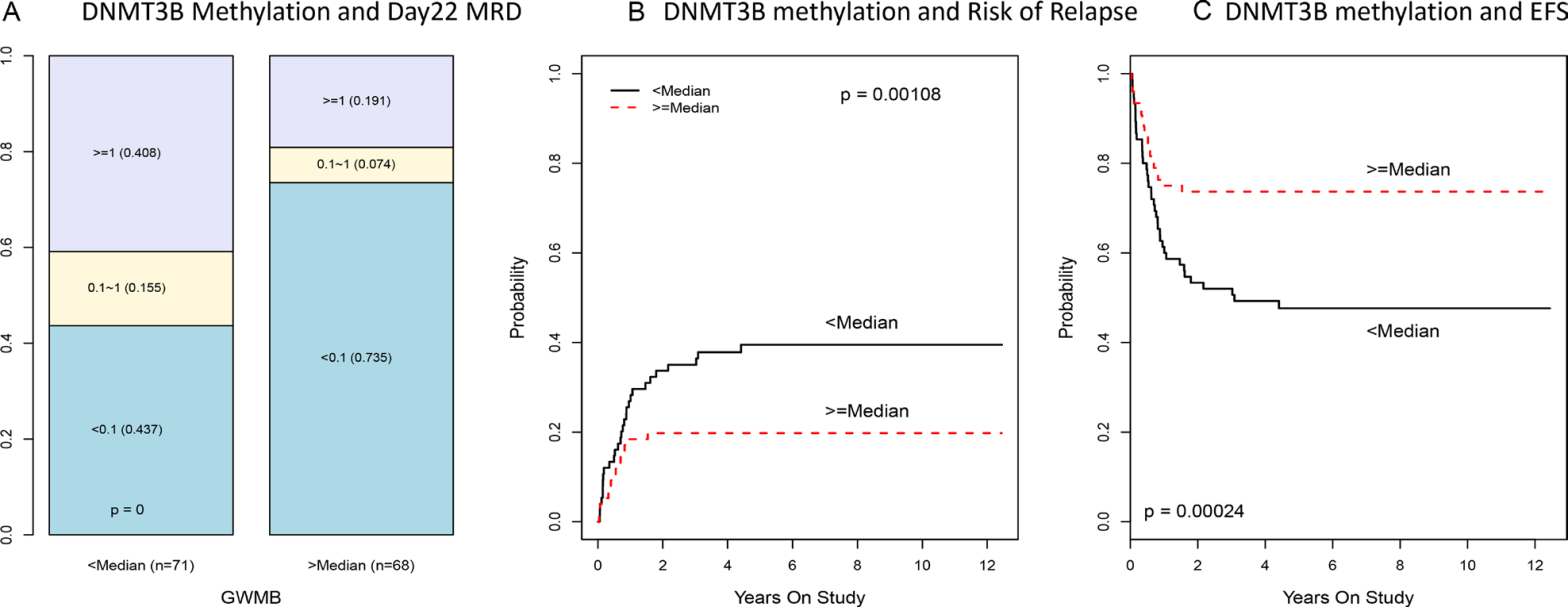
Association of DNMT3B methylation with MRD, risk of relapse and EFS in multicenter AML02 cohort (**A**) Boxplot of DNMT3B methylation score by day 22 MRD status. (**B**) The cumulative incidence of risk of relapse and resistance disease (RR) in pediatric AML according to DNMT3B methylation score. (**C**) The cumulative incidence of event free survival (EFS) in pediatric AML according to DNMT3B methylation score.

**Figure 3:**
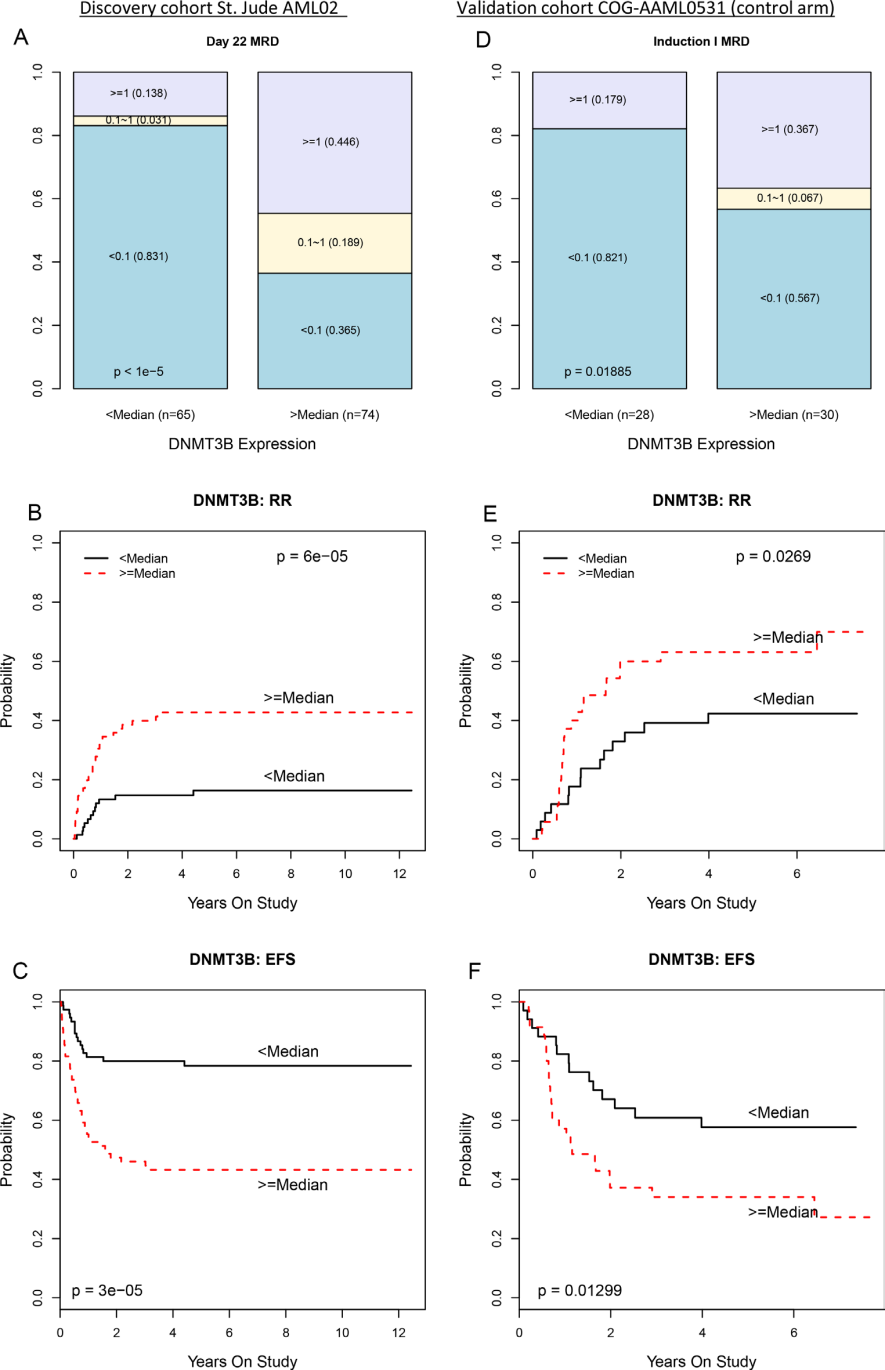
Greater DNMT3B expression associates with worse clinical outcomes in the multicenter AML02 and COG-AAML0531 clinical trials (**A**) Stacked bar plot showing proportion of AML02 patients who were MRD negative (blue), had MRD < 1% (yellow), or MRD > 1% (purple) with less than median DNMT3B expression (left column) or greater than median DNMT3B expression (right column). (**B**) Cumulative incidence of resistant disease or relapse among AML02 patients with less than median DNMT3B expression (black line) or greater than median DNMT3B expression (dashed red line). (**C**) Kaplan-Meier estimates of EFS for AML02 patients with low DNMT3B expression (black line) and high DNMT3B expression (dashed red line). Panels (**D**), (**E**), and (**F**) provide analogous results for AAML0531 (control arm) patients.

We further investigated DNA methyltransferase 3B (DNMT3B) due to its epigenetic relevance, high level of statistical significance in our analysis, the relevance of DNA methylation alterations in adult AML, and well-known biological function as a DNA methyltransferase that can potentially alter the methylation and expression of many other genes. (Table 1 and Figure 1). The methylation and expression of DNMT3B showed a strong negative association (CC, *R* = −0.74; *p* < 10^–13^; *q* < 10^–11^; Supplementary Figure 2) indicating possible epigenetic regulation. Reduced methylation and increased expression of DNMT3B associated with worse clinical outcomes (CC-PROMISE *p* < 0.00001; *q* < 0.002, Supplementary Figure 2). More specifically, reduced DNMT3B methylation associated with a greater rate of minimal residual disease (MRD, *p* < 0.00001; *q* < 0.002; Figure 2A), a greater rate of relapse or resistant disease (RR, *p* = 0.001; *q* = 0.06; Figure 2B), and worse event free survival (EFS, *p* = 0.00024; *q* = 0.03; Figure 2C). Also, concordant with the negative association observed between DNMT3B methylation and its expression, greater DNMT3B expression was associated with a greater MRD (*p* < 0.00001; *q* = 0.01; Figure 3A) a greater RR (*p* = 0.00006; *q* = 0.06; Figure 3B) and worse EFS (*p* = 0.00003; *q* = 0.04; Figure 3C). These results are consistent with two adult studies showing that greater DNMT3B expression associates with worse outcome in adult AML [32, 33]. Even though we observed both DNMT3B expression (*p* = 1.610^–10^; Supplementary Figure 3A) and DNMT3B methylation (*p* = 910^–13^; Supplementary Figure 3B) to be associated with risk group, the associations of DNMT3B expression, DNMT3B methylation, and clinical outcomes are not entirely attributable to the association of clinical risk group as these associations remained significant after adjusting for risk group (CC-PROMISE, *p* = 0.023). In particular, greater DNMT3B expression remained associated with greater MRD (*p* = 0.037) and greater RR (*p* = 0.060) in the multicenter AML02 cohort after adjusting for clinical risk group. Additionally, the association between DNMT3B methylation and expression was consistent within low-risk patients (*R* = 0.42, *p* = 0.0028), standard risk patients (*R* = −0.80, *p* < 10–15), and high-risk patients (R = −0.41, *p* = 0.0072). Wide-variability observed in both methylation scores and gene expression levels in standard risk group patients, prompted us to evaluate DNMT3B and consistent with the results for the whole cohort, an increased DNMT3B expression score and lower DNMT3B methylation score showed association with inferior outcome within this group (Supplementary Figure 4).

### DNMT3B expression is also a poor prognostic factor in COG-AAML0531 cohort

The associations of DNMT3B expression with MRD and RR observed in the AML02 discovery cohort were confirmed in a validation cohort of similarly treated patients from the control treatment arm of the COG-AAML0531 clinical trial [34] (NCT00372593). Microarray gene expression data is publicly available from these patients through the TARGET project (https://ocg.cancer.gov/programs/target). Within the AAML0531 validation cohort, greater DNMT3B expression associated with greater MRD (*p* = 0.019, Figure 3D), worse RR (*p* = 0.027, Figure 3E) and worse EFS (*p* = 0.013, Figure 3F).

### DNMT3B is an epigenetic master regulator of transcription in pediatric AML

As DNA methyltransferases are involved in de novo DNA methylation [35], DNMT3B may epigenetically modulate the methylation and the expression of other genes. Our data indicates that DNMT3B expression may profoundly impact methylation and transcription across the entire pediatric AML genome. In the multicenter AML02 discovery cohort, DNMT3B expression associates very significantly with 51,554 methylation markers (*q* < 0.01, Supplementary Figure 5A) and expression of 811 genes (*q* < 0.01, Supplementary Figure 5B). In the AAML0531 validation cohort, DNMT3B expression associated significantly with the expression of 860 genes (*q* = 0.01, Supplementary Figure 5C). We identified with 223 unique genes to be significantly associated with DNMT3B in both datasets. These striking results strongly suggest that DNMT3B may epigenetically regulate the expression levels of many genes by regulating methylation levels. Furthermore, in the AML02 cohort, DNMT3B expression associated with the expression of 41 of the 50 genes associated with clinical outcome in the initial CC-PROMISE analysis (*p* ≤ 0.05; Supplementary Table 3 and Supplementary Figure 6); suggesting that DNMT3B may epigenetically regulate the expression of these genes that may be especially relevant to AML prognosis. Three consensus sequences of unclear biological relevance were identified among the methylation probe sequences of these genes that most strongly correlated with DNMT3B expression (Supplementary Table 4).

### Genome-wide methylation burden (GWMB) is an indicator of poor prognosis

**Figure 4:**
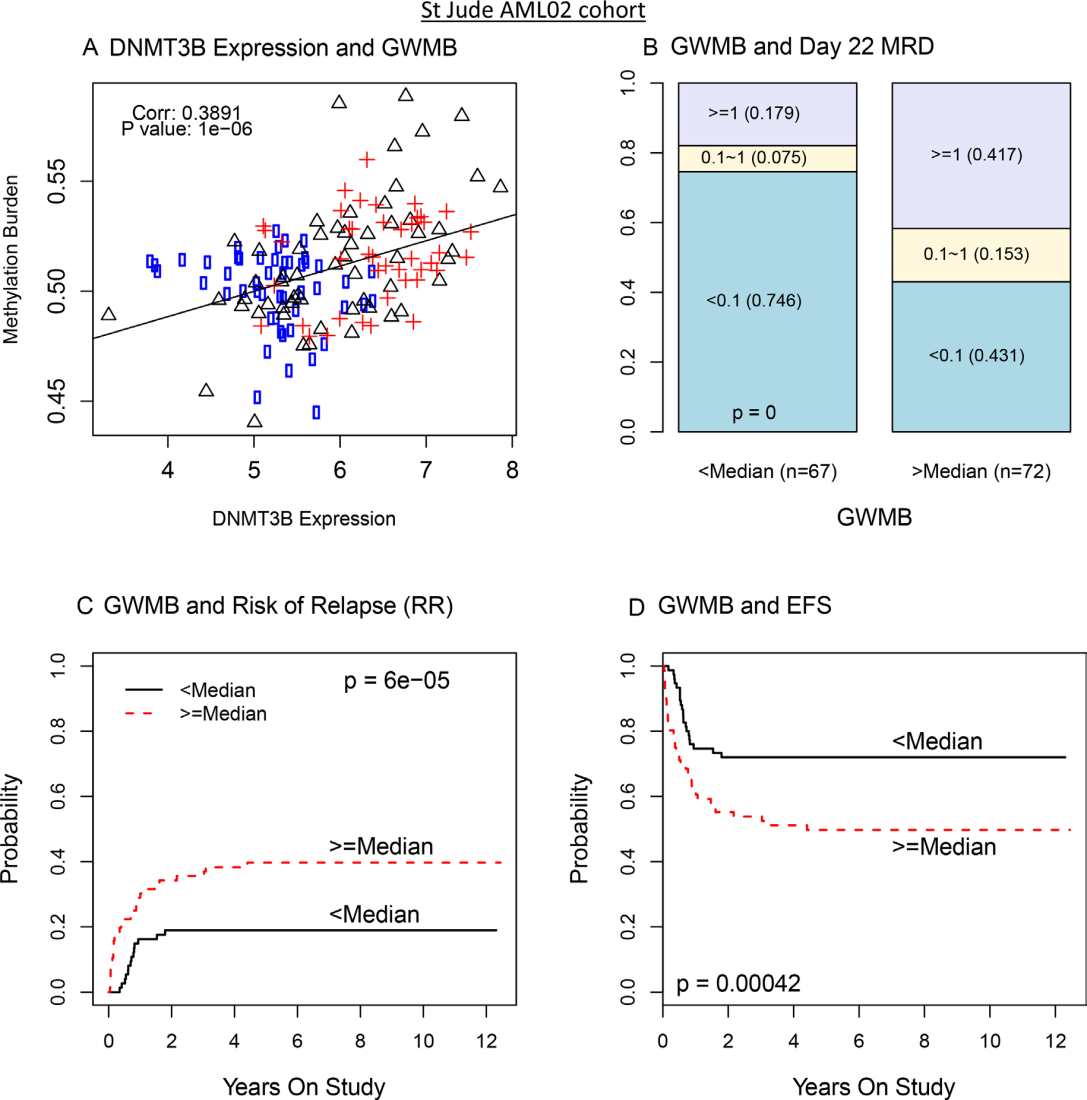
DNMT3B expression impacts genome wide methylation burden (GWMB) and GWMB is associated with outcome: AML02 cohort (**A**) Scatterplot of DNMT3B expression with overall methylation burden (circle: low risk; triangle: standard risk; cross: high risk). (**B**) Bar plot of MRD according to GWMB. (**C**) The cumulative incidence of risk of relapse and resistance disease in pediatric AML according to GWMB. (**D**) The cumulative incidence of EFS in pediatric AML according to GWMB.

To further characterize the epigenetic impact of DNMT3B expression, we computed the genome-wide methylation burden (GWMB) for each patient sample as the sum of methylation β values across all markers on the microarray. We then evaluated the association of GWMB with DNMT3B expression and clinical outcome. In the AML02 discovery cohort, greater GWMB was significantly associated with greater DNMT3B expression (*R* = 0.39; *p* = 10–6, Figure 4A), greater MRD rate (*p* = 0.000002; Figure 4B), a greater RR (*p* = 0.0075; Figure 4C), and worse EFS (*p* = 0.00024; Figure 4D). Similarly, in the validation cohort COG-AAML0531, among control treatment arm patients with available methylation array data, greater GWMB was significantly associated with greater DNMT3B expression (*R* = 0.32, *p* = 0.018; Supplementary Figure 7A), significantly associated with greater MRD (*p* = 0.004; Supplementary Figure 7B), concordantly associated with greater RR (*p* = 0.18; Supplementary Figure 7C), and significantly associated with worse EFS (*p* = 0.037; Supplementary Figure 7D). These results provide additional evidence suggesting that greater DNMT3B expression and greater GWMB are potential molecular markers indicative of a worse prognosis in the context of therapy similar to that of AML02 and the control therapy arm of AAML0531.

### Other DNA methyltransferases do not show consistent evidence of prognostic relevance

In our study, other DNMT genes did not consistently show strong evidence of prognostic relevance. MRD, RR, or EFS did not show significant associations with the expression of DNMT3A, DNMT3L, or DNMT1 in both the AML02 and AAML0531 cohorts (Supplementary Figures 8–10). No significant associations of DNMT3A expression or DNMT3L expression with MRD, RR, or EFS were observed (all *p* > 0.075, Supplementary Figures 8–9). Greater expression of DNMT1 was significantly associated with worse RR (*p* = 0.033) and EFS (*p* = 0.019) but not MRD (*p* = 0.997) in AAML0531; however, there was no evidence of such an association in AML02 (all *p* > 0.43; Supplementary Figure 9).

## DISCUSSION

DNA methylation plays a central role in self renewal and differentiation of normal hematopoietic stem cells and other cells [36–38]. CpG methylation regulates the myeloid vs. lymphoid fates by changing the methylation and thus expression levels of the genes of significant importance in lineage specific differentiation [36]. Previous studies have shown that methylation is involved in the biology and outcomes of AML in adults [9, 15]. In adult AML, recurrent genetic mutations have been observed in epigenetic regulators such as DNMT3A, TET2, IDH1, IDH2, ASXL2, EZH2, and WT1 [14]. However, mutations in these genes are very rare or nonexistent in pediatric AML [16]. Even though in NPM1 and DNMT3A are frequently mutated in adult AML, DNMT3A has been suggested as a follow-up marker [39], NPM1 and DNMT3A are each rarely mutated in pediatric AML [16, 40]. Given the distinct biology and clinical outcomes of AML in adults vs. pediatric AML and epigenomic differences in adult and pediatric population, there is an unmet need for understanding the prognostic significance of DNA methylation in pediatric AML.

**Figure 5:**
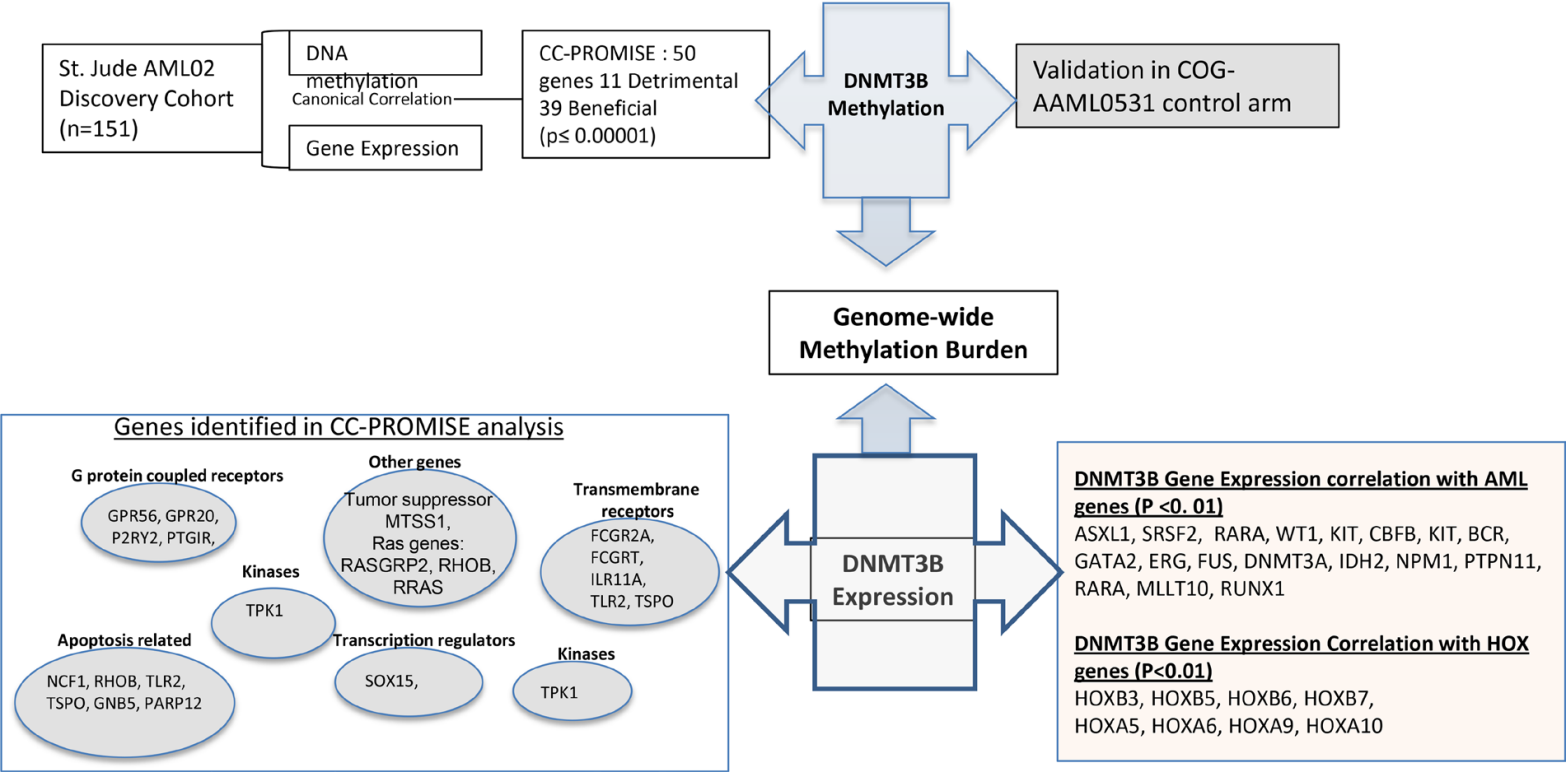
Overview of the current study Integrated analysis of genome-wide methylation and gene- expression of leukemia cells obtained at diagnosis from patients enrolled in multicenter AML02 study identified 50 genes of significant prognostic value. DNMT3B is one of the top genes identified. Methylation of DNMT3B is potential regulator of own expression, and DNMT3B expression regulated genome-wide methylation burden, both of which are predictors of clinical outcome (AML02 study and validation in AAML0531). DNMT3B is correlated with expression and methylation levels of key candidate genes involved in various cellular pathways, which in turn are potential contributors to AML outcome. Also are shown genes of significance in AML as well as HOX family of genes with significant expression correlation with expression levels of DNMT3B.

In this study, we report the first integrated and comprehensive genome-wide evaluation of DNA methylation and mRNA expression profiles with clinical outcome. Our integrated analysis identified 50 genes with epigenetic-transcriptomic signature predictive of clinical outcome. Our results clearly indicate that methylation is an important component of the biology and prognosis of pediatric AML. Using the data from two separate cohorts, we show that methylation of DNMT3B associates with its own expression and the methylation and expression of many genes across the entire genome of pediatric AML; including the genes that associate with clinical outcomes in AML02, suggesting a possible role of DNMT3B in regulating these genes, which requires further *in vitro* validation. Greater DNMT3B expression associates with increased methylation and reduced mRNA expression levels of MTSS1, consistent with a recent report showing that MTSS1 expression is regulated by DNA methylation and more specifically being targeted by DNMT3B [19, 20, 41]. MTSS1 has a potential tumor suppressor function, which may explain the association of greater expression (and lesser methylation) of MTSS1 with reduced MRD22 and reduced risk of relapse. GPR56, G protein –coupled receptor, has been identified as a molecular signature of leukemic stem cells in AML [22, 31]. High expression of GPR56 has been associated with HOXA9 induced-leukemogenesis in mice [21, 22]. Within EV1-high AML, GPR56 has been shown to have high expression as well as higher antiapoptotic activities, which is consistent with our results. MS4A3, performs an anti-proliferative role by inhibiting G1-S cell cycle transition, perhaps through the CDK signaling pathway [42]. A recent study has shown that agonistic targeting of TLR1/TLR2 induces apoptosis and differentiation of AML cells, which may represent a new therapeutic strategy for AML. Lastly, MT3 a metallothionein III, has been identified as a tumor suppressor in various cancers as well as in pediatric AML [29]. Down regulation of MT3 has been implicated in metastasis and poor clinical outcome in solid tumors. Also, MT3 promoter hypermethylation has been associated with reduced MT3 expression and apoptosis in AML cells [29]. We also observed significant correlation of DNMT3B expression levels with 17 previously reported genes of significance in AML [14]. Among the HOX family of genes, which have been shown to have significant relevance in AML, we observed positive correlation of DNMT3B expression with HOXB3, HOXB6, HOXB7, HOXA5, HOXA6, HOXA9 and HOXA10. This suggests that epigenetic alteration of the DNMT3B locus may be a contributor to transcriptional deregulation that potentially contributes to the development of pediatric AML. Figure 5 summarizes genes identified in this study and those of importance in AML with significant correlation with DNMT3B expression in childhood AML.

Our results show DNMT3B methylation and expression levels to be predictive of clinical outcome measured by MRD after induction 1, risk of relapse and event free survival in childhood AML. This is consistent with two previous reports that greater DNMT3B expression associated with worse outcome in adult AML [33, 32]. A recent report by Larmonie *et al.*, 2017, identified differential methylation of CpG sites within MN1 oncogene between pediatric AML patients with or without inv [16, 43]. Interestingly within this study, only DNMT3B expression and not any other DNMTs (DNMT3A or DNMT1) were inversely associated with MN1 gene expression, thereby indicating a potential role of DNMT3B mediated regulation of MN1 in inv [16] AML. These results suggest that differences in DNMT3B methylation or expression may not only characterize biological differences between different AML risk groups and hence the process of leukemogenesis, but may also characterize other processes that associate with prognosis among standard risk patients. Although the focus of this manuscript is not subgroup discovery, consistent with this report, we observed a significant correlation of DNMT3B with MN1 expression in both AML02 and COG-AAML0531 cohorts.

In summary, we report the first integrated DNA methylation and gene expression analysis in pediatric AML patients. Our results suggest a significant contribution of DNMT3B. DNMT3B has been shown to play a role in the restoration of methylation post treatment with DNA hypomethylating agents such as azacytidine [44]. In addition to having a methyl-transferase activity, DNMT3B also acts as an accessory protein in complex with DNMT3A contributing to aberrant methylation in cancer. Although more work is needed to understand the complex biology of DNMTs, current evidence suggests that DNMT3A has a lower capability of restoring methylation post DNMT-inhibitor (DNMTi) treatment without involvement of DNMT3B [44]. Thus expression levels of DNMT3B might be critical in remethylation post DNMTi treatment.

Our results suggest two possible conceptual models of how DNMT3B and total methylation may impact AML development and prognosis. DNMT3B may act as a master epigenetic regulator of the transcriptome that activates pathways that promote AML development and/or suppresses pathways that inhibit AML development. Also, DNMT3B expression may activate pathways that promote drug resistance or suppress pathways that promote drug sensitivity. Increased total methylation may promote resistance to cytarabine if methylcytosine interferes with the incorporation of this cytosine analog into genomic DNA. Nucleoside analogs such as azacytidine and decitabine that result in global hypomethylation have shown encouraging results in MDS and AML. Although these DNMTis are not specific for any DNMTs, our results showing higher GWMB that corresponds with higher level of DNMT3B suggests reversal via DNMTis might improve treatment outcome in pediatric AML.

## MATERIALS AND METHODS

### Patient population

Patients treated on the multi-center AML02 clinical trial (NCT00136084) were included in this study. Details of study design and clinical outcome are described elsewhere [45]. Briefly, patients were randomized to receive high (3 g/m^2^, given every 12 h on days 1, 3 and 5) or low dose (100 mg/m^2^ given every 12 h on days 1–10) cytarabine along with daunorubicin and etoposide as a first course of chemotherapy with subsequent treatment tailored to response and risk classification. Of the 232 patients enrolled in the study, we were able to obtain quality both transcriptome and methylome data for 151 subjects. Patient characteristics are provided in Supplementary Table 1. There were no differences in clinical outcome endpoints between patients included in the present analysis vs. that were not part of this analysis Supplementary Table 1. St. Jude Institutional Review Board approved the study, and informed consent was obtained from parents/guardians and consents/assents from the individuals.

### Methylation profiling

Bone marrow was obtained at diagnosis and genomic DNA was isolated from leukemic blasts using DNA blood kit (Qiagen, CA, USA). Samples had >80% leukemic cells or they were enriched to achieve >80% blasts by immunomagnetic sorting [Miltenyi Biotech, Germany] prior to DNA isolation. Bisulfide conversion of genomic DNA was performed using Zymo EZ DNA Methylation kit (Zymo Research). Bisulfite converted DNA was hybridized to Illumina 450 K methylation beadchip arrays (Illumina) as per manufacturers instruction at University of Minnesota, Biomedical Genomics Center. Hybridization fluorescent signals were read using Illumina Beat Station GX scanner. All samples achieved greater than 95% call rate. The data was controlled for batch effects as described in the Supplementary Methods.

### Gene expression profiling

Gene expression profiling of leukemic blasts obtained at diagnosis was performed using GeneChip^®^ Human Genome U133 Plus 2.0 Array [Affymetrix, Santa Clara, CA]. Details regarding RNA isolation, labeling of cRNA, and scanning of Affymetrix arrays have been published previously [46]. The MAS 5.0 algorithm was used to obtain normalized gene expression signals.

### Clinical outcomes

AML02 patients with t[8;21], inv[16], or t[9;11] chromosome abnormalities were classified as having low-risk AML. High-risk AML classification included those with -7, *FLT3*-ITD mutation, t[6;9], megakaryoblastic AML, treatment-related AML, or AML arising from MDS. Patients lacking low or high-risk group features were classified as standard-risk AML. Details of clinical response criteria and outcome are provided elsewhere [45]. Overall minimal residual disease (MRD at day 22) was measured using flow cytometry after the first course of chemotherapy as previously described [47]. MRD was defined as 1 or more AML cells per 1000 bone marrow mononuclear cells [i.e., ≥ 0.1%]. Event-Free survival (EFS) was defined as the time elapsed from study enrollment to the earliest of death, disease resistance, relapse, or second malignancy with event-free subjects censored at last follow-up. We defined the time to relapse or resistant (RR) disease as the time from study enrollment to disease resistance or relapse with subjects not having these events censored at date of last follow-up or death in remission. Patient characteristics and details of endpoints are provided in Supplementary Table 1.

### Statistical methods

Section 1 of the Supplementary Methods describes the preprocessing of microarray data for the AML02 cohort.

Canonical correlation with projection onto the most interesting statistical evidence (CC-PROMISE; [18]) was used to perform an integrated analysis of the association of the methylation and expression of each gene with MRD and RR. Spearman's method was used to evaluate the association of MRD as an ordinal variable with expression and methylation. The method of Jung, Owzar, and George [48] was used to evaluate the association of RR and EFS with expression and methylation. *P*-values were computed by 100,000 permutations of the assignment of clinical outcome data to the molecular data. Additional details about the CC-PROMISE analysis are available in section 2 of the Supplementary Methods. For each set of *p*-values from these analyses, robust estimates of the false discovery rate (FDR) were obtained and reported with *q*-value nomenclature [49]. The same methods were used to evaluate the associations of genome-wide methylation burden (GWMB) with other variables but no multiplicity adjustments were performed for this small number of tests. Spearman's correlation was used to evaluate the association of the DNMT3B methylation and expression scores obtained by CC-PROMISE for each risk group.

Associations among DNMT3B expression, GWMB, and clinical outcomes observed in the AML02 discovery cohort were also evaluated in a validation cohort of 132 pediatric patients receiving similar therapy on the standard arm of the AAML0531 clinical trial [34] with data publicly available from the TARGET project (https://ocg.cancer.gov/programs/target). Section 3 of the Supplementary Methods provides additional details about the validation analyses using data from patients enrolled in standard arm of COG_AAML0531. All reported *p*-values are two-sided.

## SUPPLEMENTARY MATERIALS FIGURES AND TABLES



## References

[1] Jiang D, Hong Q, Shen Y, Xu Y, Zhu H, Li Y, Xu C, Ouyang G, Duan S (2014). The diagnostic value of DNA methylation in leukemia: a systematic review and meta-analysis. PLoS One.

[2] Kim TK, Gore SD, Zeidan AM (2015). Epigenetic Therapy in Acute Myeloid Leukemia: Current and Future Directions. Semin Hematol.

[3] Alvarez S, Suela J, Valencia A, Fernandez A, Wunderlich M, Agirre X, Prosper F, Martin-Subero JI, Maiques A, Acquadro F, Rodriguez Perales S, Calasanz MJ, Roman-Gomez J (2010). DNA methylation profiles and their relationship with cytogenetic status in adult acute myeloid leukemia. PLoS One.

[4] Brunetti L, Gundry MC, Goodell MA (2017). DNMT3A in Leukemia. Cold Spring Harb Perspect Med.

[5] Claus R, Plass C, Armstrong SA, Bullinger L (2010). DNA methylation profiling in acute myeloid leukemia: from recent technological advances to biological and clinical insights. Future Oncol.

[6] Marcucci G, Yan P, Maharry K, Frankhouser D, Nicolet D, Metzeler KH, Kohlschmidt J, Mrozek K, Wu YZ, Bucci D, Curfman JP, Whitman SP, Eisfeld AK (2014). Epigenetics meets genetics in acute myeloid leukemia: clinical impact of a novel seven-gene score. J Clin Oncol.

[7] Plass C, Oakes C, Blum W, Marcucci G (2008). Epigenetics in acute myeloid leukemia. Semin Oncol.

[8] Li Y, Xu Q, Lv N, Wang L, Zhao H, Wang X, Guo J, Chen C, Li Y, Yu L (2017). Clinical implications of genome-wide DNA methylation studies in acute myeloid leukemia. J Hematol Oncol.

[9] Figueroa ME, Lugthart S, Li Y, Erpelinck-Verschueren C, Deng X, Christos PJ, Schifano E, Booth J, van Putten W, Skrabanek L, Campagne F, Mazumdar M, Greally JM (2010). DNA methylation signatures identify biologically distinct subtypes in acute myeloid leukemia. Cancer Cell.

[10] Akalin A, Garrett-Bakelman FE, Kormaksson M, Busuttil J, Zhang L, Khrebtukova I, Milne TA, Huang Y, Biswas D, Hess JL, Allis CD, Roeder RG, Valk PJ (2012). Base-pair resolution DNA methylation sequencing reveals profoundly divergent epigenetic landscapes in acute myeloid leukemia. PLoS Genet.

[11] Bullinger L, Ehrich M, Dohner K, Schlenk RF, Dohner H, Nelson MR, van den Boom D (2010). Quantitative DNA methylation predicts survival in adult acute myeloid leukemia. Blood.

[12] Deneberg S, Grovdal M, Karimi M, Jansson M, Nahi H, Corbacioglu A, Gaidzik V, Dohner K, Paul C, Ekstrom TJ, Hellstrom-Lindberg E, Lehmann S (2010). Gene-specific and global methylation patterns predict outcome in patients with acute myeloid leukemia. Leukemia.

[13] Saied MH, Marzec J, Khalid S, Smith P, Down TA, Rakyan VK, Molloy G, Raghavan M, Debernardi S, Young BD (2012). Genome wide analysis of acute myeloid leukemia reveal leukemia specific methylome and subtype specific hypomethylation of repeats. PLoS One.

[14] Grimwade D, Ivey A, Huntly BJ (2016). Molecular landscape of acute myeloid leukemia in younger adults and its clinical relevance. Blood.

[15] Prada-Arismendy J, Arroyave JC, Rothlisberger S (2017). Molecular biomarkers in acute myeloid leukemia. Blood Rev.

[16] Ho PA, Kutny MA, Alonzo TA, Gerbing RB, Joaquin J, Raimondi SC, Gamis AS, Meshinchi S (2011). Leukemic mutations in the methylation-associated genes DNMT3A and IDH2 are rare events in pediatric AML: a report from the Children's Oncology Group. Pediatr Blood Cancer.

[17] Alisch RS, Barwick BG, Chopra P, Myrick LK, Satten GA, Conneely KN, Warren ST (2012). Age-associated DNA methylation in pediatric populations. Genome Res.

[18] Cao X, Crews KR, Downing J, Lamba J, Pounds SB (2016). CC-PROMISE effectively integrates two forms of molecular data with multiple biologically related endpoints. BMC Bioinformatics.

[19] Fan H, Chen L, Zhang F, Quan Y, Su X, Qiu X, Zhao Z, Kong KL, Dong S, Song Y, Chan TH, Guan XY (2012). MTSS1, a novel target of DNA methyltransferase 3B, functions as a tumor suppressor in hepatocellular carcinoma. Oncogene.

[20] Schemionek M, Kharabi Masouleh B, Klaile Y, Krug U, Hebestreit K, Schubert C, Dugas M, Buchner T, Wormann B, Hiddemann W, Berdel WE, Brummendorf TH, Muller-Tidow C (2015). Identification of the Adapter Molecule MTSS1 as a Potential Oncogene-Specific Tumor Suppressor in Acute Myeloid Leukemia. PLoS One.

[21] Daria D, Kirsten N, Muranyi A, Mulaw M, Ihme S, Kechter A, Hollnagel M, Bullinger L, Dohner K, Dohner H, Feuring-Buske M, Buske C (2016). GPR56 contributes to the development of acute myeloid leukemia in mice. Leukemia.

[22] Pabst C, Bergeron A, Lavallee VP, Yeh J, Gendron P, Norddahl GL, Krosl J, Boivin I, Deneault E, Simard J, Imren S, Boucher G, Eppert K (2016). GPR56 identifies primary human acute myeloid leukemia cells with high repopulating potential *in vivo*. Blood.

[23] Schulze I, Rohde C, Scheller-Wendorff M, Baumer N, Krause A, Herbst F, Riemke P, Hebestreit K, Tschanter P, Lin Q, Linhart H, Godley LA, Glimm H (2016). Increased DNA methylation of Dnmt3b targets impairs leukemogenesis. Blood.

[24] Ishihara M, Araya N, Sato T, Tatsuguchi A, Saichi N, Utsunomiya A, Nakamura Y, Nakagawa H, Yamano Y, Ueda K (2013). Preapoptotic protease calpain-2 is frequently suppressed in adult T-cell leukemia. Blood.

[25] Kikuyama M, Takeshima H, Kinoshita T, Okochi-Takada E, Wakabayashi M, Akashi-Tanaka S, Ogawa T, Seto Y, Ushijima T (2012). Development of a novel approach, the epigenome-based outlier approach, to identify tumor-suppressor genes silenced by aberrant DNA methylation. Cancer Lett.

[26] Yi JM, Dhir M, Guzzetta AA, Iacobuzio-Donahue CA, Heo K, Yang KM, Suzuki H, Toyota M, Kim HM, Ahuja N (2012). DNA methylation biomarker candidates for early detection of colon cancer. Tumour Biol.

[27] Dunne J, Cullmann C, Ritter M, Soria NM, Drescher B, Debernardi S, Skoulakis S, Hartmann O, Krause M, Krauter J, Neubauer A, Young BD, Heidenreich O (2006). siRNA-mediated AML1/MTG8 depletion affects differentiation and proliferation-associated gene expression in t(8;21)-positive cell lines and primary AML blasts. Oncogene.

[28] Heller G, Rommer A, Steinleitner K, Etzler J, Hackl H, Heffeter P, Tomasich E, Filipits M, Steinmetz B, Topakian T, Klingenbrunner S, Ziegler B, Spittler A (2015). EVI1 promotes tumor growth via transcriptional repression of MS4A3. J Hematol Oncol.

[29] Tao YF, Xu LX, Lu J, Cao L, Li ZH, Hu SY, Wang NN, Du XJ, Sun LC, Zhao WL, Xiao PF, Fang F, Li YH (2014). Metallothionein III (MT3) is a putative tumor suppressor gene that is frequently inactivated in pediatric acute myeloid leukemia by promoter hypermethylation. J Transl Med.

[30] Steinbach D, Bader P, Willasch A, Bartholomae S, Debatin KM, Zimmermann M, Creutzig U, Reinhardt D, Gruhn B (2015). Prospective validation of a new method of monitoring minimal residual disease in childhood acute myelogenous leukemia. Clin Cancer Res.

[31] Steinbach D, Schramm A, Eggert A, Onda M, Dawczynski K, Rump A, Pastan I, Wittig S, Pfaffendorf N, Voigt A, Zintl F, Gruhn B (2006). Identification of a set of seven genes for the monitoring of minimal residual disease in pediatric acute myeloid leukemia. Clin Cancer Res.

[32] Hayette S, Thomas X, Jallades L, Chabane K, Charlot C, Tigaud I, Gazzo S, Morisset S, Cornillet-Lefebvre P, Plesa A, Huet S, Renneville A, Salles G (2012). High DNA methyltransferase DNMT3B levels: a poor prognostic marker in acute myeloid leukemia. PLoS One.

[33] Niederwieser C, Kohlschmidt J, Volinia S, Whitman SP, Metzeler KH, Eisfeld AK, Maharry K, Yan P, Frankhouser D, Becker H, Schwind S, Carroll AJ, Nicolet D (2015). Prognostic and biologic significance of DNMT3B expression in older patients with cytogenetically normal primary acute myeloid leukemia. Leukemia.

[34] Gamis AS, Alonzo TA, Meshinchi S, Sung L, Gerbing RB, Raimondi SC, Hirsch BA, Kahwash SB, Heerema-McKenney A, Winter L, Glick K, Davies SM, Byron P (2014). Gemtuzumab ozogamicin in children and adolescents with de novo acute myeloid leukemia improves event-free survival by reducing relapse risk: results from the randomized phase III Children's Oncology Group trial AAML0531. J Clin Oncol.

[35] Okano M, Bell DW, Haber DA, Li E (1999). DNA methyltransferases Dnmt3a and Dnmt3b are essential for de novo methylation and mammalian development. Cell.

[36] Benetatos L, Vartholomatos G (2018). Enhancer DNA methylation in acute myeloid leukemia and myelodysplastic syndromes. Cell Mol Life Sci.

[37] Moore LD, Le T, Fan G (2013). DNA methylation and its basic function. Neuropsychopharmacology.

[38] Shahid M, Gull N, Yeon A, Cho E, Bae J, Yoon HS, You S, Yoon H, Kim M, Berman BP, Kim J (2018). Alpha-oxoglutarate inhibits the proliferation of immortalized normal bladder epithelial cells via an epigenetic switch involving ARID1A. Sci Rep.

[39] Alpermann T, Schnittger S, Eder C, Dicker F, Meggendorfer M, Kern W, Schmid C, Aul C, Staib P, Wendtner CM, Schmitz N, Haferlach C, Haferlach T (2016). Molecular subtypes of NPM1 mutations have different clinical profiles, specific patterns of accompanying molecular mutations and varying outcomes in intermediate risk acute myeloid leukemia. Haematologica.

[40] Mullighan CG, Kennedy A, Zhou X, Radtke I, Phillips LA, Shurtleff SA, Downing JR (2007). Pediatric acute myeloid leukemia with NPM1 mutations is characterized by a gene expression profile with dysregulated HOX gene expression distinct from MLL-rearranged leukemias. Leukemia.

[41] Utikal J, Gratchev A, Muller-Molinet I, Oerther S, Kzhyshkowska J, Arens N, Grobholz R, Kannookadan S, Goerdt S (2006). The expression of metastasis suppressor MIM/MTSS1 is regulated by DNA methylation. Int J Cancer.

[42] Donato JL, Ko J, Kutok JL, Cheng T, Shirakawa T, Mao XQ, Beach D, Scadden DT, Sayegh MH, Adra CN (2002). Human HTm4 is a hematopoietic cell cycle regulator. J Clin Invest.

[43] Larmonie NSD, Arentsen-Peters TCJM, Obulkasim A, Valerio D, Sonneveld E, Danen-van Oorschot AA, de Haas V, Reinhardt D, Zimmermann M, Trka J, Baruchel A, Pieters R, van den Heuvel-Eibrink MM (2018). MN1 overexpression is driven by loss of DNMT3B methylation activity in inv(16) pediatric AML. Oncogene.

[44] Duymich CE, Charlet J, Yang X, Jones PA, Liang G (2016). DNMT3B isoforms without catalytic activity stimulate gene body methylation as accessory proteins in somatic cells. Nat Commun.

[45] Rubnitz JE, Inaba H, Dahl G, Ribeiro RC, Bowman WP, Taub J, Pounds S, Razzouk BI, Lacayo NJ, Cao X, Meshinchi S, Degar B, Airewele G (2010). Minimal residual disease-directed therapy for childhood acute myeloid leukaemia: results of the AML02 multicentre trial. Lancet Oncol.

[46] Ross ME, Mahfouz R, Onciu M, Liu HC, Zhou X, Song G, Shurtleff SA, Pounds S, Cheng C, Ma J, Ribeiro RC, Rubnitz JE, Girtman K (2004). Gene expression profiling of pediatric acute myelogenous leukemia. Blood.

[47] Coustan-Smith E, Ribeiro RC, Rubnitz JE, Razzouk BI, Pui CH, Pounds S, Andreansky M, Behm FG, Raimondi SC, Shurtleff SA, Downing JR, Campana D (2003). Clinical significance of residual disease during treatment in childhood acute myeloid leukaemia. Br J Haematol.

[48] Jung SH, Owzar K, George SL (2005). A multiple testing procedure to associate gene expression levels with survival. Stat Med.

[49] Pounds S, Cheng C (2006). Robust estimation of the false discovery rate. Bioinformatics.

