# Correction: Integrated epigenetic and genetic analysis identifies markers of prognostic significance in pediatric acute myeloid leukemia

**DOI:** 10.18632/oncotarget.25792

**Published:** 2018-07-13

**Authors:** Jatinder K. Lamba, Xueyuan Cao, Susana C. Raimondi, Roya Rafiee, James R. Downing, Lei Shi, Tanja Gruber, Raul C. Ribeiro, Jeffrey E. Rubnitz, Stanley B. Pounds

**Affiliations:** ^1^ Department of Pharmacotherapy and Translational Research, Center for Pharmacogenomics, University of Florida, Gainesville, FL, USA; ^2^ Department of Biostatistics, St. Jude Children's Research Hospital, Memphis, TN, USA; ^3^ Department of Pathology, St. Jude Children's Research Hospital, Memphis, TN, USA; ^4^ Department of Oncology, St. Jude Children's Research Hospital, Memphis, TN, USA

**This article has been corrected:** The corrected author name is given below:

**Lei Shi**

Original article: Oncotarget. 2018; 9:26711-26723. https://doi.org/10.18632/oncotarget.25475

