# Supplementary material for: Integrated epigenetic and genetic analysis identifies markers of prognostic significance in pediatric acute myeloid leukemia
Source: Oncotarget. 2018 Jun 1;9(42):26711–23. doi: 10.18632/oncotarget.25475 (PMC6003565; doi:10.18632/oncotarget.25475)
Supplement: Supplementary file 1 [file oncotarget-09-26711-s001.pdf]

Integrated epigenetic and genetic analysis identifies markers of prognostic significance in pediatric acute myeloid leukemia

SUPPLEMENTARY MATERIALS

SUPPLEMENTARY METHODS

Preprocessing of microarray data

Affymetrix U133A microarray expression data for the AML02 cohort were pre-processed by the MAS5.0 algorithm and log-transformed. Illumina 450 K methylation array data were pre-processed with the subset within array normalization (SWAN; [1]) as implemented in the minfi R package [2]. The methylation profiling was carried out in 5 batches. Out of the 151 samples, 147 detected at least 95% loci, 2 detected 90% loci, and the other 2 detected 94% loci. To adjust for batch effect, M values were modeled in a linear regression with batch as the independent predictor. The residuals were treated as batch adjusted M values.

CC-PROMISE analysis

The CC-PROMISE method was used to identify candidate genes based on the correlation of methylation and expression with each other and their pattern of association with minimal residual disease and risk of relapse. The Affymetrix annotation was used to annotate each expression probe-set to a gene. For each gene, canonical correlation was used to evaluate the association of expression with methylation, noting that expression and methylation may each be measured by multiple probe-sets in each gene. Classically, the sign of the canonical correlation statistic is arbitrary. To enhance biological interpretation, in this study we assigned the sign of the canonical correlation statistic to match that of the univariate correlation of the average expression with the average methylation. Also, canonical correlation empirically defines an expression score and a methylation score that were further evaluated for association with minimal residual disease and risk of relapse or resistant disease as described below.

We used Spearman's correlation to associate the canonical correlation methylation score and the canonical correlation expression score with minimal residual disease ordinally categorized as undetected (or negative), between 0.1–1%, and >1%. We used the rank-based statistic for censored time-to-event variables of Jung, Owzar, and George (Jung *et al*, 2005 [3]) to evaluate the association of the methylation score and the expression score with relapse. In the JOG statistic, we defined the time to relapse as the time from study enrollment to disease resistance or relapse with subjects not having these events censored at date of last follow-up or death in remission. The PROMISE statistic was defined as a linear combination of the association of the CC expression score with MRD, association of CC expression score with risk of relapse, association of the CC methylation score with MRD, and the association of CC methylation score with risk of relapse using the Spearman and JOG statistics. The absolute value of all coefficients in this linear combination was one. The sign of the coefficients were defined as described below.

The PROMISE statistic was defined so that a positive sign indicated that greater expression was associated with better clinical outcomes (i.e. reduced risk of relapse and reduced levels of MRD). The signs of the coefficients for the association of the methylation score were defined to match those of the canonical correlation statistics as described above. In short, the PROMISE statistic was defined to identify a concordant pattern of associations among expression, methylation, MRD and risk of relapse as shown below:

PR (M,X,MRD,RR) = –(Sp (X,MRD) + JOG (X,RR) + sign (Spearman (M*,X*)) (Sp(M,MRD) + JOG(M,RR)))

where M is the CC methylation score, X is the CC expression score, MRD is the ordinal MRD (undetected, 0.1–1%, >1%), RR is rate of relapse or resistant disease, M* is the average M-value across methylation probes annotated to the gene, and X* is the average expression level across expression probe-sets annotated to the gene. All association statistics were computed on a correlation scale ranging from –1 to +1.

Validation of associations in the AAML0531 cohort

The AML TARGET project (https://ocg.cancer.gov/programs/target) has made clinical, methylation, and expression data publicly available for a subcohort of patients treated on the AAML0531 and AAML03P1 clinical trials performed by the Children's Oncology Group. Gemtuzumab ozogamicin (GO) was included in two courses of chemotherapy for all patients on the

AAML03P1 trial. Patients on the AAML0531 trial were randomized to receive GO during two courses of chemotherapy on the experimental arm or no GO on the control therapy arm. Also, the AAML0531 trial found that GO significantly improved EFS. In the AML02 trial, the use of GO was very limited; a few patients with poor response to induction chemotherapy received one dose of GO. All trials were similar with respect to administration of other drugs. Thus, we chose to use the control therapy arm of the AAML0531 trial as a validation cohort for our study.

The TARGET project has made clinical outcome and microarray expression available for 69 subjects on the control arm of AAML0531. We used this cohort of patients to test the association of DNMT3B expression with clinical outcomes: MRD, RR, EFS. We performed one-sided tests to improve power of this limited cohort to confirm the associations observed in AML02. The results of these analyses are shown in Figure 2 of the primary manuscript.

For these 69 subjects, 68 had 27 K methylation array data and 3 had 450 K methylation array data. Due to the limited number of subjects with 450 K array data (same platform used in the AML02 study), we used the cohort of 68 patients with both microarray expression data and 27 K methylation array data to evaluate the association of genome-wide methylation burden with DNMT3B expression. The result of this analysis is shown in Supplementary Figure 7A.

A cohort of 53 AAML0531 control arm subjects had 450 K methylation array data available. The control-therapy arm subjects with data available were not representative of the association of risk group with clinical outcome. The publicly reported outcomes for control-arm subjects on the trial as a whole are a three year EFS of 64.0% for low risk disease, 45.8% for intermediate risk disease, and 27.2% for high-risk disease. However, for the 53 subjects with publicly available 450 K methylation array data, 7 of 8 (87%) low-risk patients, 35 of 42 (83%) intermediate risk, and 8 of 10 (80%) high risk patients experienced an EFS event. Due to the reverse ordering of risk groups by outcome among this cohort of patients (low risk was worse than intermediate risk which was worse than high risk) with 450 K methylation array data, we chose to use the cohort of 42 intermediate risk patients to validate the association of GWMB with clinical outcomes that was observed in AML02. The results of that analysis are shown in Supplementary Figures S7b, S7c, and S7d. The tests are one-sided to improve power of this limited cohort to confirm the associations observed in AML02.

We also examined the association of 450 K array GWMB with clinical outcome within the low-risk and high-risk subjects (Supplementary Figure 8). In each case, we observed the trend of greater GWMB associating with worse clinical outcomes, but the associations were not statistically significant with these very limited sample sizes.

REFERENCES

1. Maksimovic J, Gordon L, Oshlack A. SWAN: Subset-quantile within array normalization for illumina infinium HumanMethylation450 BeadChips. Genome Biol. 2012; 13:R44. https://doi.org/10.1186/gb-2012-13-6-r44.
2. Aryee MJ, Jaffe AE, Corrada-Bravo H, Ladd-Acosta C, Feinberg AP, Hansen KD, Irizarry RA. Minfi: a flexible and comprehensive Bioconductor package for the analysis of Infinium DNA methylation microarrays. Bioinformatics. 2014; 30:1363–9. https://doi.org/10.1093/bioinformatics/btu049.
3. Jung SH, Owzar K, George SL. A multiple testing procedure to associate gene expression levels with survival. Stat Med. 2005; 24:3077–88.

Supplementary Table 1: Summary of patient characteristics (St. Jude AML02 cohort) that were included in the present study

Charateristics	Level	Patients included in the study	Patients excluded due to lack of specimen availability	*P* value
Treatment arm	HDAC	70	43	0.060
	LDAC	81	36	
	Not randomized	0	2	
Gender	Female	70	32	0.328
	Male	81	49	
Age group	<10	76	45	0.499
	≥10	75	36	
Age (continuous)		9.262 (0.0137~20.11)	5.837 (0.1697~21.35)	0.495
Provisional risk	High	43	33	**0.044**
	Low	50	19	
	Standard	58	29	
WBC Group	<50	104	66	**0.046**
	≥50	47	15	
WBC (continuous)		24.6 (0.013~513.6)	11 (0.017~409)	**0.002**
Race	Black	26	17	0.903
	Other	17	8	
	Unknown	1	1	
	White	107	55	
Cytogenetic group	11q23	20	7	0.093
	Insuff	2	4	
	inv (16)	20	6	
	Miscell	40	33	
	Normal	37	16	
	t (8;21)	23	8	
	t (9;11)	9	7	
Day 22 MRD	Inevaluable	10	10	0.209
	Negative	83	47	
	Positive	58	24	
Induction I response		0	2	0.275
	CR	116	64	
	NR	18	5	
	PR	17	10	
Induction II response		8	7	0.556
	CR	135	72	
	NR	1	1	
	PR	7	1	
Event-free survival	5 years	0.607 ± 0.04	0.564 ± 0.055	0.597
Overall survival	5 years	0.724 ± 0.037	0.653 ± 0.055	0.2887

Statistical Methods for Supplementary Table 1: The Cochran-Armitage trend test was used to evaluate associations of specimen availability with provisional risk group, induction I response, and induction II response as ordinal variables. The log-rank test was used to evaluate associations of specimen availability with event-free survival and overall survival. The rank-sum test was used to evaluate the association of WBC as a quantitative continuous variable with specimen availability. Fisher's exact test was used to evaluate all other associations.

Characteristics of patients that were part of the parent clinical trial but were not included in the present analysis is also shown.

Supplementary Table 2: Results of risk stratified CC-PROMISE analysis of the 50 candidate genes with methylation and expression associated with clinical outcome

Association variables	Expression				Methylation				CC-PROMISE Integrated analysis	
	MRD		RR		MRD		RR			
Gene	R	*p*	R	*p*	R	*p*	R	*p*	R	*p*
ALDH3A1	–0.1623	0.0568	–0.1780	0.0266	0.2348	0.0054	0.1972	0.0135	0.1931	0.0005
BLVRA	–0.1343	0.1140	–0.2181	0.0061	0.2189	0.0095	0.2223	0.0058	0.1984	0.0007
BLVRA	–0.1343	0.1140	–0.2181	0.0061	0.2189	0.0095	0.2223	0.0058	0.1984	0.0007
CALML4	–0.1070	0.2108	–0.1883	0.0178	0.0606	0.4758	0.1251	0.1218	0.1203	0.0329
CAPN2	–0.2092	0.0137	–0.1199	0.1371	0.1739	0.0402	0.1854	0.0210	0.1721	0.0071
CHST12	0.1299	0.1276	0.1508	0.0626	–0.1721	0.0437	–0.1159	0.1510	–0.1422	0.0133
COTL1	–0.1534	0.0721	–0.0275	0.7335	0.3335	0.0001	0.1211	0.1341	0.1589	0.0078
DNMT3B	0.1759	0.0369	0.1514	0.0598	–0.1865	0.0283	–0.0269	0.7407	–0.1352	0.0230
FBN2	–0.1422	0.0959	–0.2592	0.0012	0.1212	0.1562	0.2349	0.0033	0.1894	0.0016
FCGR2A	–0.1546	0.0678	–0.1230	0.1266	0.1145	0.1784	0.1718	0.0325	0.1410	0.0073
FCGRT	–0.0652	0.4463	–0.1123	0.1675	0.1406	0.0991	0.0770	0.3415	0.0988	0.0893
GNB5	0.1780	0.0367	0.2881	0.0003	0.1516	0.0750	0.2585	0.0010	–0.2190	0.0001
GPR20	–0.0701	0.4108	–0.1896	0.0180	0.1235	0.1488	0.2486	0.0018	0.1580	0.0051
GPR56	0.0719	0.4010	0.2100	0.0093	0.0231	0.7892	0.0348	0.6684	–0.0849	0.1309
GRPEL1	–0.1636	0.0547	–0.1361	0.0910	–0.2009	0.0176	–0.1939	0.0147	0.1737	0.0013
HYAL2	–0.1165	0.1721	–0.2620	0.0008	0.0338	0.6912	0.1916	0.0165	0.1510	0.0088
LSP1	0.1230	0.1488	0.2058	0.0103	0.1999	0.0177	0.2169	0.0066	–0.1864	0.0019
LSP1	0.1230	0.1488	0.2058	0.0103	0.1999	0.0177	0.2169	0.0066	–0.1864	0.0019
LSP1	0.1230	0.1488	0.2058	0.0103	0.1999	0.0177	0.2169	0.0066	–0.1864	0.0019
MS4A3	–0.0936	0.2727	–0.2079	0.0091	0.0755	0.3763	0.3027	0.0002	0.1699	0.0041
MSLN	–0.2475	0.0031	0.0071	0.9305	0.2151	0.0104	0.0541	0.5011	0.1274	0.0308
MTSS1	–0.1209	0.1570	–0.2235	0.0054	0.1951	0.0209	0.3017	0.0001	0.2103	0.0004
NCF1	–0.2766	0.0011	–0.1017	0.2087	0.1985	0.0193	0.1395	0.0820	0.1791	0.0006
NCF1	–0.2766	0.0011	–0.1017	0.2087	0.1985	0.0193	0.1395	0.0820	0.1791	0.0006
NCF1	–0.2766	0.0011	–0.1017	0.2087	0.1985	0.0193	0.1395	0.0820	0.1791	0.0006
PARP12	–0.2626	0.0016	–0.1297	0.1068	0.1772	0.0365	0.2147	0.0073	0.1961	0.0003
PPP1R1A	0.0054	0.9496	–0.1244	0.1234	0.2015	0.0170	0.1448	0.0724	0.1163	0.0243
PRG2	–0.1448	0.0889	–0.3398	0.0000	0.1234	0.1473	0.2556	0.0013	0.2159	0.0000
PTGIR	–0.0467	0.5856	–0.1877	0.0193	0.0441	0.6026	0.2086	0.0091	0.1218	0.0372
RNH1	–0.0934	0.2751	–0.1585	0.0496	0.1045	0.2217	0.1931	0.0160	0.1374	0.0212
SOX15	–0.0403	0.6381	–0.1445	0.0734	0.1112	0.1929	0.2346	0.0034	0.1327	0.0146
SPINK2	0.1526	0.0717	0.1845	0.0220	–0.1170	0.1716	–0.2089	0.0090	–0.1657	0.0069
TARP	0.2509	0.0029	0.0402	0.6204	–0.2515	0.0026	–0.0697	0.3909	–0.1531	0.0082
TLR2	–0.0685	0.4237	–0.2518	0.0015	0.0102	0.9046	0.2848	0.0003	0.1538	0.0080
TPK1	–0.1021	0.2318	–0.1857	0.0208	0.1973	0.0199	0.1203	0.1354	0.1513	0.0065
TPSB2	0.0280	0.7443	–0.2422	0.0024	0.1519	0.0738	0.0649	0.4221	0.1077	0.0128
TPSB2	0.0280	0.7443	–0.2422	0.0024	0.1519	0.0738	0.0649	0.4221	0.1077	0.0128
TPSD1	–0.1248	0.1412	–0.2064	0.0092	0.0656	0.4426	0.1861	0.0200	0.1457	0.0089
USP20	0.1336	0.1164	0.1193	0.1395	0.2287	0.0068	0.1556	0.0530	–0.1593	0.0036
CRIP2	–0.0489	0.5661	–0.0594	0.4642	0.1061	0.2150	0.1895	0.0176	0.1010	0.0711
EPX	–0.0788	0.3538	–0.2019	0.0113	0.1061	0.2151	0.1487	0.0641	0.1339	0.0101
GLB1L	0.0143	0.8660	–0.1155	0.1512	0.2417	0.0039	0.1504	0.0605	0.1233	0.0270
GPX7	–0.0180	0.8307	–0.0280	0.7291	0.0749	0.3813	0.1423	0.0762	0.0658	0.2409
IL11RA	0.0756	0.3760	0.1230	0.1270	0.1930	0.0227	0.2145	0.0070	–0.1515	0.0046
KDELR3	0.1167	0.1715	0.2151	0.0067	–0.3024	0.0003	–0.2291	0.0040	–0.2158	0.0000
LAMB2	0.0216	0.8006	–0.1656	0.0387	0.0909	0.2859	0.1848	0.0205	0.1049	0.0754
LPL	–0.1597	0.0599	–0.2562	0.0011	0.2439	0.0040	0.2801	0.0003	0.2350	0.0000
MT3	–0.0170	0.8431	–0.2004	0.0124	0.1370	0.1077	0.1414	0.0791	0.1240	0.0178
P2RY2	–0.1285	0.1324	–0.0783	0.3334	0.1828	0.0311	0.1516	0.0604	0.1353	0.0231

Supplementary Table 3: Association of DNMT3B expression with gene expression and top 3 methylation probes of the 50 candidate genes identified in outcome CC-PROMISE analysis (Table 1)

Gene	Gene expression correlationStat	*Gene expression correlation. P value*	Methylation probe1. correlation. stat	*Methylation probe1. correlation. P value*	Methylation probe2. correlation. Stat	*Methylation probe2. correlation.P value*	Methylation probe3. correlation. Stat	*Methylation probe3. correlation. P value*
DNMT3B	1	*0*	-0.4966	*8.85E-11*	0.4927	*1.31E-10*	0.4926	*1.33E-10*
CHST12	0.5977	*4.44E-16*	-0.3890	*7.99E-07*	–0.3760	*1.96E-06*	–0.3513	*9.76E-06*
SPINK2	0.5124	*1.76E-11*	-0.3871	*9.09E-07*	–0.1240	*0.1292*	0.1153	*0.1585*
TARP	0.3564	*7.08E-06*	-0.2850	*0.0004*	–0.0748	*0.3614*	0.0622	*0.4482*
PARP12	–0.2125	*0.0088*	0.2007	*0.0135*	0.1250	*0.1263*	0.1016	*0.2145*
RASGRP2	0.1656	*0.0421*	0.2435	*0.0026*	0.2033	*0.0123*	0.1871	*0.0215*
MS4A3	–0.2083	*0.0103*	0.2706	*0.0008*	0.2385	*0.0032*	0.1841	*0.0236*
TPSB2	–0.1061	*0.1946*	0.2771	*0.0006*	NA	*NA*	NA	*NA*
TPK1	–0.4382	*1.84E-08*	0.3015	*0.0002*	0.2856	*0.0004*	0.2106	*0.0094*
PRG2	–0.0630	*0.4419*	0.3096	*0.0001*	0.2076	*0.0105*	0.1608	*0.0486*
GNB5	0.3716	*2.63E-06*	0.3112	*0.0001*	0.2730	*0.0007*	0.1762	*0.0304*
TLR2	–0.4636	*2.05E-09*	0.3135	*8.85E-05*	0.3030	*0.0002*	0.2758	*0.0006*
LPL	–0.1847	*0.0232*	0.3165	*7.51E-05*	0.3104	*0.0001*	0.2677	*0.0009*
GPR56	0.5054	*3.62E-11*	0.3167	*7.42E-05*	0.2785	*0.0005*	0.2601	*0.0013*
SCO2	–0.3577	*6.52E-06*	0.3388	*2.08E-05*	0.2463	*0.0023*	0.2452	*0.0024*
FBN2	–0.2350	*0.0037*	0.3394	*2.01E-05*	0.2792	*0.0005*	0.2412	*0.0029*
GLB1L	–0.1290	*0.1143*	0.3396	*1.99E-05*	0.2647	*0.0010*	0.1935	*0.0173*
NCF1	–0.3790	*1.60E-06*	0.3469	*1.28E-05*	–0.0367	*0.6545*	NA	*NA*
ALDH3A1	–0.1237	*0.1304*	0.3553	*7.58E-06*	0.3128	*9.24E-05*	0.2507	*0.0019*
PTGIR	–0.2547	*0.0016*	0.3562	*7.15E-06*	0.3444	*1.49E-05*	0.3180	*6.93E-05*
USP20	0.4956	*9.78E-11*	0.3565	*7.01E-06*	0.2303	*0.0044*	0.2181	*0.0071*
GPX7	–0.3049	*0.0001*	0.3613	*5.18E-06*	0.3603	*5.52E-06*	0.3544	*8.02E-06*
FCGRT	–0.5303	*2.51E-12*	0.3655	*3.93E-06*	0.3377	*2.22E-05*	0.2480	*0.0021*
RRAS	–0.2881	*0.0003*	0.3868	*9.33E-07*	0.2396	*0.0030*	0.1928	*0.0177*
IL11RA	0.3123	*9.45E-05*	0.3981	*4.13E-07*	0.2089	*0.0101*	0.2078	*0.0105*
MTSS1	–0.4165	*1.04E-07*	0.4030	*2.89E-07*	0.3632	*4.56E-06*	0.3108	*0.0001*
P2RY2	–0.4404	*1.53E-08*	0.4038	*2.73E-07*	0.3725	*2.48E-06*	0.3692	*3.08E-06*
SLC11A1	–0.4065	*2.22E-07*	0.4143	*1.24E-07*	0.3790	*1.59E-06*	0.3690	*3.13E-06*
GRPEL1	–0.3847	*1.07E-06*	0.4173	*9.83E-08*	–0.1819	*0.0254*	0.1725	*0.0342*
CAPN2	–0.5038	*4.29E-11*	0.4181	*9.21E-08*	0.3978	*4.24E-07*	0.3768	*1.85E-06*
TPSD1	–0.1161	*0.1558*	0.4229	*6.33E-08*	0.3545	*7.97E-06*	0.3439	*1.53E-05*
TSPO	–0.4908	*1.57E-10*	0.4279	*4.23E-08*	0.3269	*4.18E-05*	0.3224	*5.41E-05*
BLVRA	–0.4974	*8.18E-11*	0.4390	*1.72E-08*	0.4066	*2.21E-07*	0.3971	*4.47E-07*
TPSAB1	–0.1180	*0.1491*	0.4428	*1.24E-08*	0.2939	*0.0002*	0.2186	*0.0070*
EPX	–0.1612	*0.0481*	0.4433	*1.19E-08*	0.4263	*4.81E-08*	0.3801	*1.48E-06*
RNH1	–0.4906	*1.61E-10*	0.4503	*6.58E-09*	0.4384	*1.80E-08*	0.4383	*1.81E-08*
RHOB	–0.2295	*0.0046*	0.4558	*4.09E-09*	0.3852	*1.04E-06*	0.3651	*4.04E-06*
HYAL2	–0.2132	*0.0086*	0.4586	*3.19E-09*	0.4346	*2.47E-08*	0.4335	*2.70E-08*
MT3	–0.1123	*0.1697*	0.4675	*1.43E-09*	0.2803	*0.0005*	0.2667	*0.0009*
PPP1R1A	–0.0378	*0.6449*	0.4682	*1.35E-09*	0.4218	*6.91E-08*	0.3596	*5.75E-06*
COTL1	–0.5959	*6.95E-16*	0.4741	*7.83E-10*	0.4668	*1.53E-09*	0.4369	*2.04E-08*
FCGR2A	–0.4526	*5.37E-09*	0.4838	*3.10E-10*	0.4631	*2.14E-09*	0.2200	*0.0066*
KDELR3	0.1412	*0.0838*	0.4893	*1.82E-10*	0.4126	*1.41E-07*	0.3273	*4.10E-05*
SOX15	–0.1965	*0.0156*	0.4977	*7.94E-11*	0.4454	*9.98E-09*	0.4108	*1.61E-07*
CALML4	–0.2740	*0.0007*	0.5241	*4.96E-12*	0.4580	*3.36E-09*	0.4151	*1.16E-07*
LAMB2	–0.2426	*0.0027*	0.5297	*2.69E-12*	0.4857	*2.59E-10*	0.4804	*4.32E-10*
LSP1	0.2391	*0.0031*	0.5302	*2.53E-12*	0.5262	*3.95E-12*	0.3933	*5.86E-07*
CRIP2	–0.2699	*0.0008*	0.5503	*2.50E-13*	0.5240	*5.01E-12*	0.4535	*5.01E-09*
MSLN	–0.4217	*6.93E-08*	0.5545	*1.51E-13*	0.5366	*1.24E-12*	0.5354	*1.41E-12*
GPR20	–0.2011	*0.0133*	0.6297	*0*	0.6151	*0*	0.5866	*2.44E-15*

Supplementary Table 4: Three motifs identified by consensus sequence search on 147 CpGs within 50 genes using MEME (Multiple Em for Motif Elicitation[#]).

1. Motif 1:

***E*-value:** 3.3e-014; **Site Count:** 9; **Width:** 41

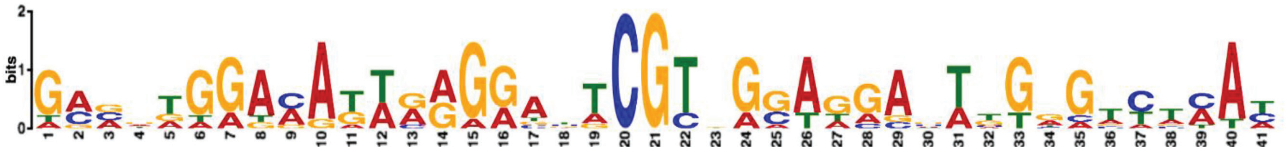

Name	Strand	Start	*p*-value		Sites	
cg08321330_TPSB2	–	2	1.52e-20	A	GAGGTGGACATTGAGGACTCGTAGGAGGACTTGGGTCTCAT	A
cg05873268_TPSAB1	+	3	1.52e-20	CA	GAGGTGGACATTGAGGACTCGTAGGAGGACTTGGGTCTCAT	
cg01375871_TPSD1	+	3	1.39e-19	CA	GAGGTGGACATTGAGGAATCGTTGGAGGACTTGGGTCTCAT	
cg01832012_TPK1	+	3	3.19e-11	AT	GAAAAAAAAATAAGGAAGTCGTGGGTTAAAAATAGCTCCAC	
cg07263235_LPL	+	3	4.81e-11	CT	GCAAGTGACAAACAGGATTCGTCAAAAGAGAGGTGTATTAA	
cg16092895_PPP1R1A	+	3	7.15e-11	GG	GGCTAGGAAAGTAGAGGGGCGCTGCAGGAGAGGAGACTAAA	
cg03892543_COTL1	–	2	4.02e-10	A	ACCATGAAAAGAAAGACCACGTGGCAGGCATCGCCATAAAC	C
cg21370481_TPK1	–	2	1.13e-9	A	GCATGGGTGATAGGGAATTCGCCACTTAGTTTGAAGCAAAT	G
cg25279100_PARP12	+	3	1.37e-9	CC	TACCTGGGAGGTAGGGTTACGTGGGAACAGTGTGGTTGATC	

2. Motif 2

***E*-value: 8.4e-004; Site Count: 4; Width: 29**

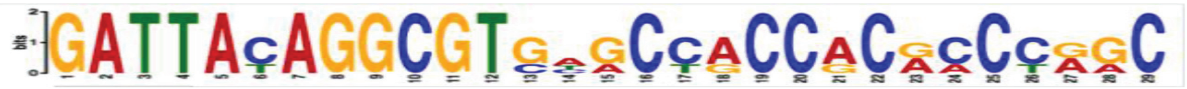

Name	Strand	Start	*p*-value		Sites	
cg27350579_EPX	+	13	8.18e-16	AAAGTGCTAG	GATTATAGGCGTGAGCCACCACACCTGGC	CC
cg06350097_FCGR2A	+	3	1.65e-15	GA	GATTACAGGCGTGAGCCACCGCGCCCGGC	CGACCAGGAA
cg15379825_TPK1	+	6	7.47e-15	GCTGG	GATTACAGGCGTGTGCCGCCACAACCAAC	TAATTTTAG
cg03311682_PTGIR	+	13	1.12e-14	GAGTAGATGA	GATTACAGGCGTCCACTACCACGCCCAGC	TA

3. Motif 3

***E*-value:** 5.4e-003; **Site Count:** 9; **Width:** 21

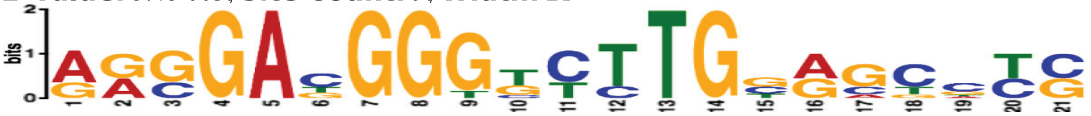

Name	Strand	Start	*p*-value		Sites	
cg08578212_TPSAB1	+	17	9.50e-11	AGGTTTCAGG	AGCGACGGGTCTTGTAGCCTG	GGGCAG
cg03466598_TPSD1	+	17	9.50e-11	AGGTTTCAGG	AGCGACGGGTCTTGTAGCCTG	GGGCAG
cg20813589_RNH1	+	5	1.71e-9	CTGT	GAGGACGGGTTTTGGAGCGCC	GCTCGGCCGT
cg01704474_RNH1	+	17	1.71e-9	GCGAGGCTGT	GAGGACGGGTTTTGGAGCGCC	GCTCGG
cg15787807_RASGRP2	+	1	1.56e-8		AGGGATGGGGCTTGCGCTCTG	CGGAGATGCT
cg06464781_RASGRP2	+	8	1.56e-8	ACGTTCT	AGGGATGGGGCTTGCGCTCTG	CGGAGATGCT
cg07719512_SLC11A1	+	20	2.02e-8	GCATTAGGCC	AACGAGGGGTCTTGGAACTCC	AGA
cg13195954_GPR20	+	17	8.78e-8	TCAGAGCCTG	AGGGACGGTGCCTGGGGCACC	AGAGTC
cg07127225_FCGRT	–	17	1.93e-7	GACGCC	GAGGAGGGGCTCTGCGGGCTC	GGTCCAGACT

[#]Timothy L. Bailey and Charles Elkan, "Fitting a mixture model by expectation maximization to discover motifs in biopolymers", Proceedings of the Second International Conference on Intelligent Systems for Molecular Biology, pp. 28-36, AAAI Press, Menlo Park, California, 1994.

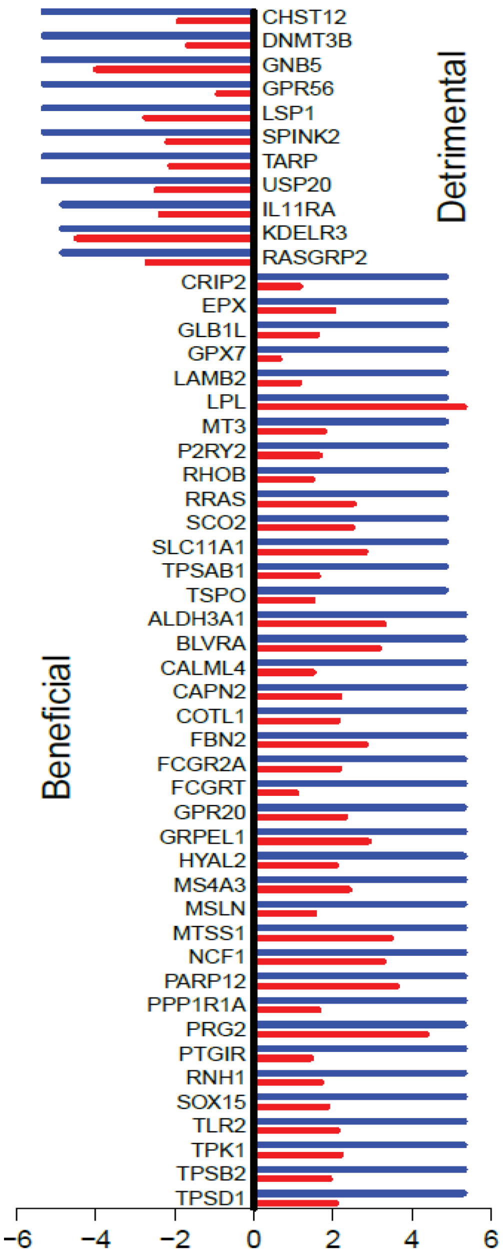

Supplementary Figure 1: Association bar plot showing the significance and direction of association of 50 genes in the CC-PROMISE analysis. The length of the bars indicates the statistical significance on the log10 *p*-value scale. The direction of the bars indicates whether greater expression of the gene associates with better clinical outcomes (beneficial) or worse clinical outcomes (detrimental). The blue bars indicate results of an unstratified CC-PROMISE analysis. The red bars provide results of a CC-PROMISE analysis that adjusts for risk group by stratification.

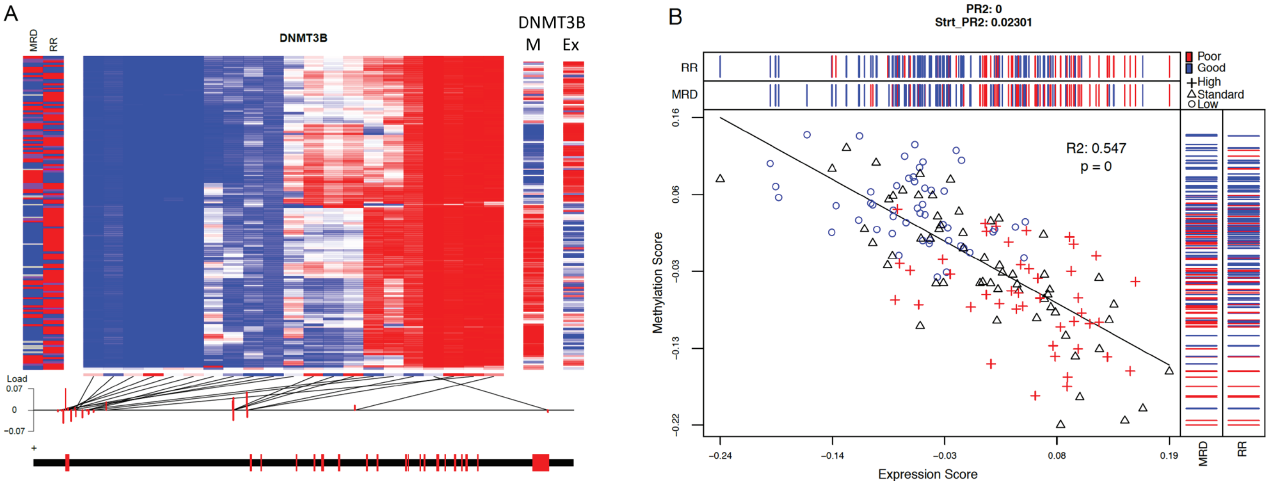

Supplementary Figure 2: Methylation and expression of DNMT3B associated with the phenotypic pa7ern defined by MRD and risk of relapse. (**A**) Heatmap of MRD, RR, methylation of several CpG sites on DNMT3B, DNMT3B-CC methylation score (M), and DNMT3B expression (Ex) in the AML02 cohort. (**B**) Scatterplot of methylation score and expression of DNMT3B. The corresponding phenotypes of MRD (minimal residual disease at day 22) and RR (risk of relapse and resistance disease) are shown in upper and left side of the scatterplot with color bars as indicated in the legend in the top right corner. Circle: low risk; Triangle: standard risk; Cross: high risk. PR2: PROMISE *p* value (from unstratified analysis); Strt_PR2 (PROMISE *p* value : risk stratified).

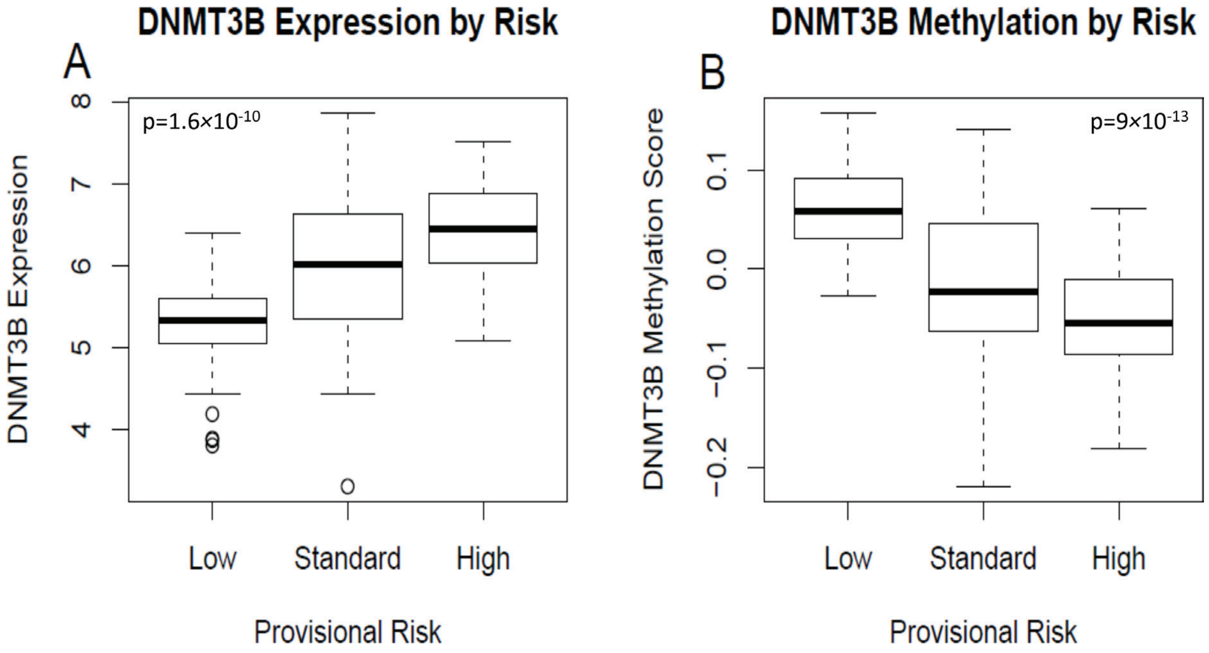

Supplementary Figure 3: (**A**) Boxplot of DNMT3B expression by clinical risk group in St. Jude AML02 cohort. (**B**) Boxplot of DNMT3B methylation by clinical risk group in St. Jude AML02 cohort.

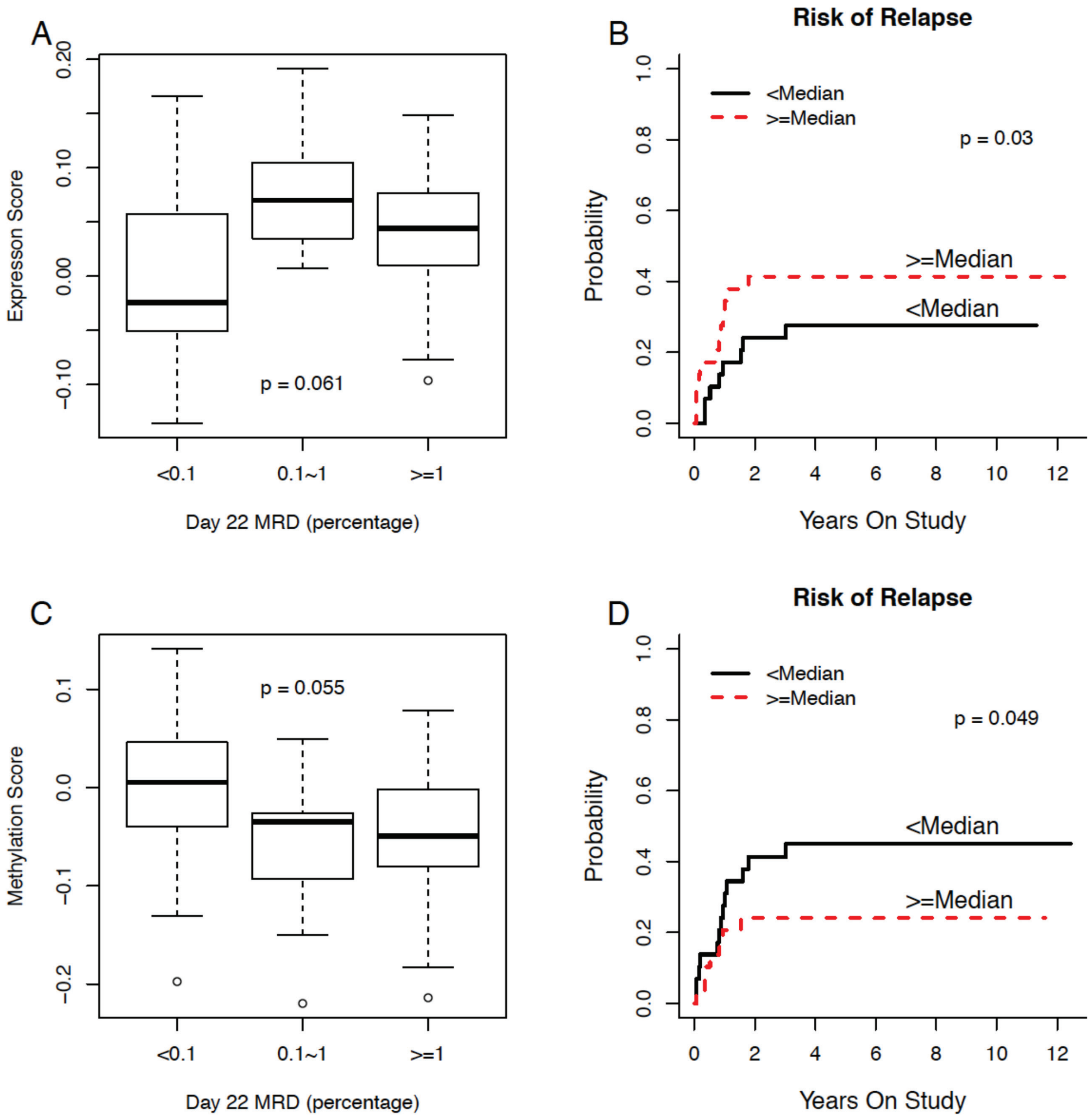

Supplementary Figure 4: DNMT3B methylation and expression of DNMT3B associated with MRD and risk of relapse in standard risk pediatric AML. Panel (**A**) shows boxplot of DNMT3B expression score by day 22 MRD status. Panel (**B**) shows the cumulative incidence of risk of relapse and resistance disease in pediatric AML according to DNMT3B expression score. Panel (**C**) shows boxplot of DNMT3B methylation score by day 22 MRD status. Panel (**D**) shows the cumulative incidence of risk of relapse and resistance disease in pediatric AML according to DNMT3B methylation score.

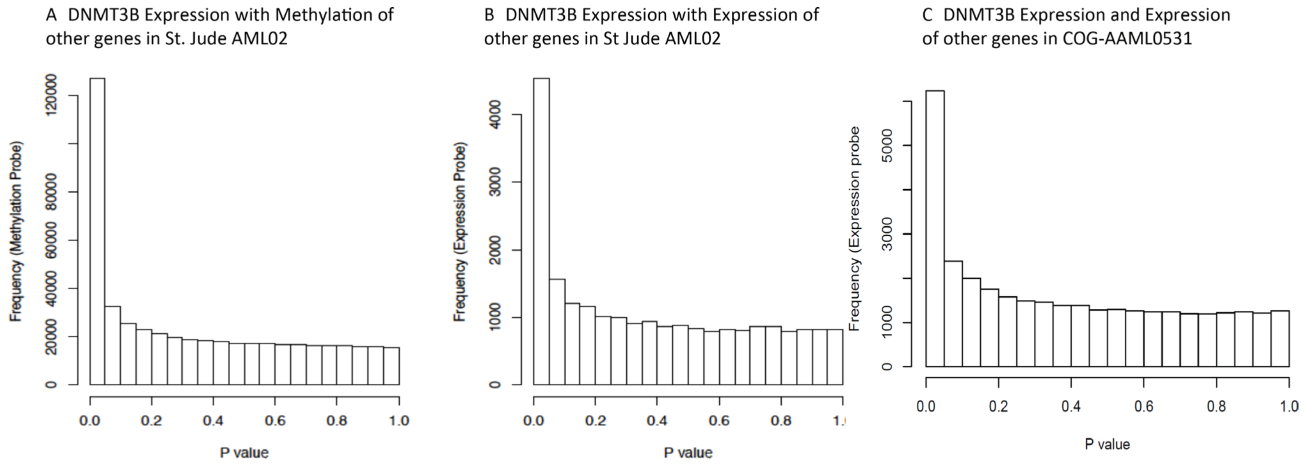

Supplementary Figure 5: Distribution of *p*-values for association DNMT3B expression with expression and methylation of other genes. (**A**) Histogram of *p*-values for association of DNMT3B expression with methylation in AML02. (**B**) Histogram of *p*-values for association of DNMT3B expression with expression of other genes in AML02. (**C**) Histogram of *p*-values for association of DNMT3B expression with expression of other genes in AAML0531. The tall mode of $p < 0.05$ indicates that many genes' expression associates with the expression of DNMT3B.

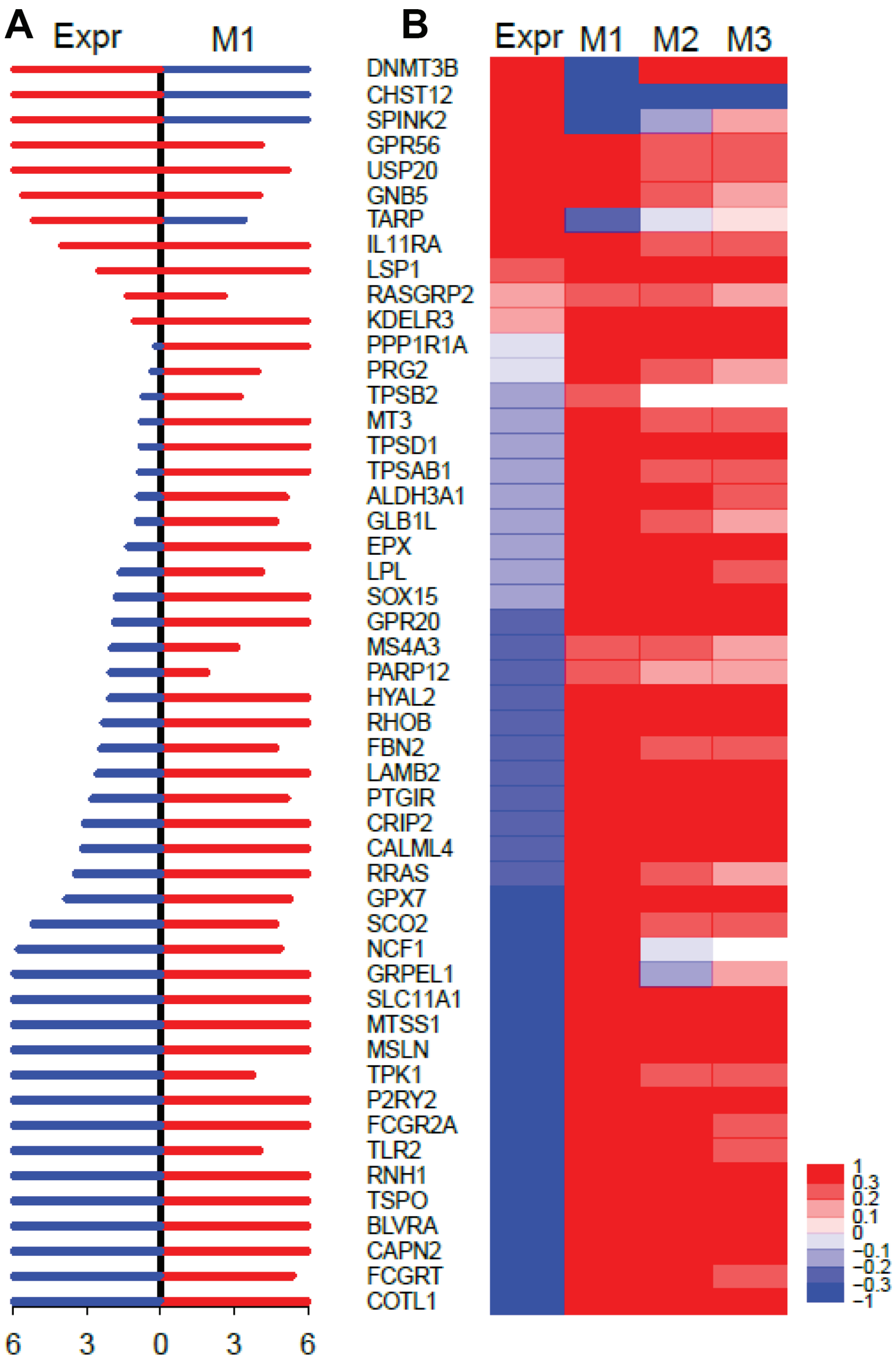

Supplementary Figure 6: Association of DNMT3B expression with expression of top 50 genes identified by CC-PROMISE analysis of AML02 data set. (**A**) Barplot of significance scores. On the left, the bars indicate the statistical significance of the association of DNMT3B with expression and methylation of 50 genes on the log10 *p*-value scale. Significance values for expression associations are shown as bars extending to the left and significance values for the most strongly associated methylation marker extend to the right. Bars shaded in blue indicate negative associations and bars shaded in red indicate positive associations. The length of the bar corresponds to the log10 *p*-value significance score as indicated by the scale at the bottom. (**B**) Association heatmap. Each block illustrates the Spearman correlation of the expression or methylation of a gene with the expression of DNMT3B. The color scale indicates the magnitude and direction of association. The E column provides the expression association result and the columns M1–M3 provide association results for the most significant methylation markers for each gene. Genes with fewer than 3 methylation markers have white empty boxes.

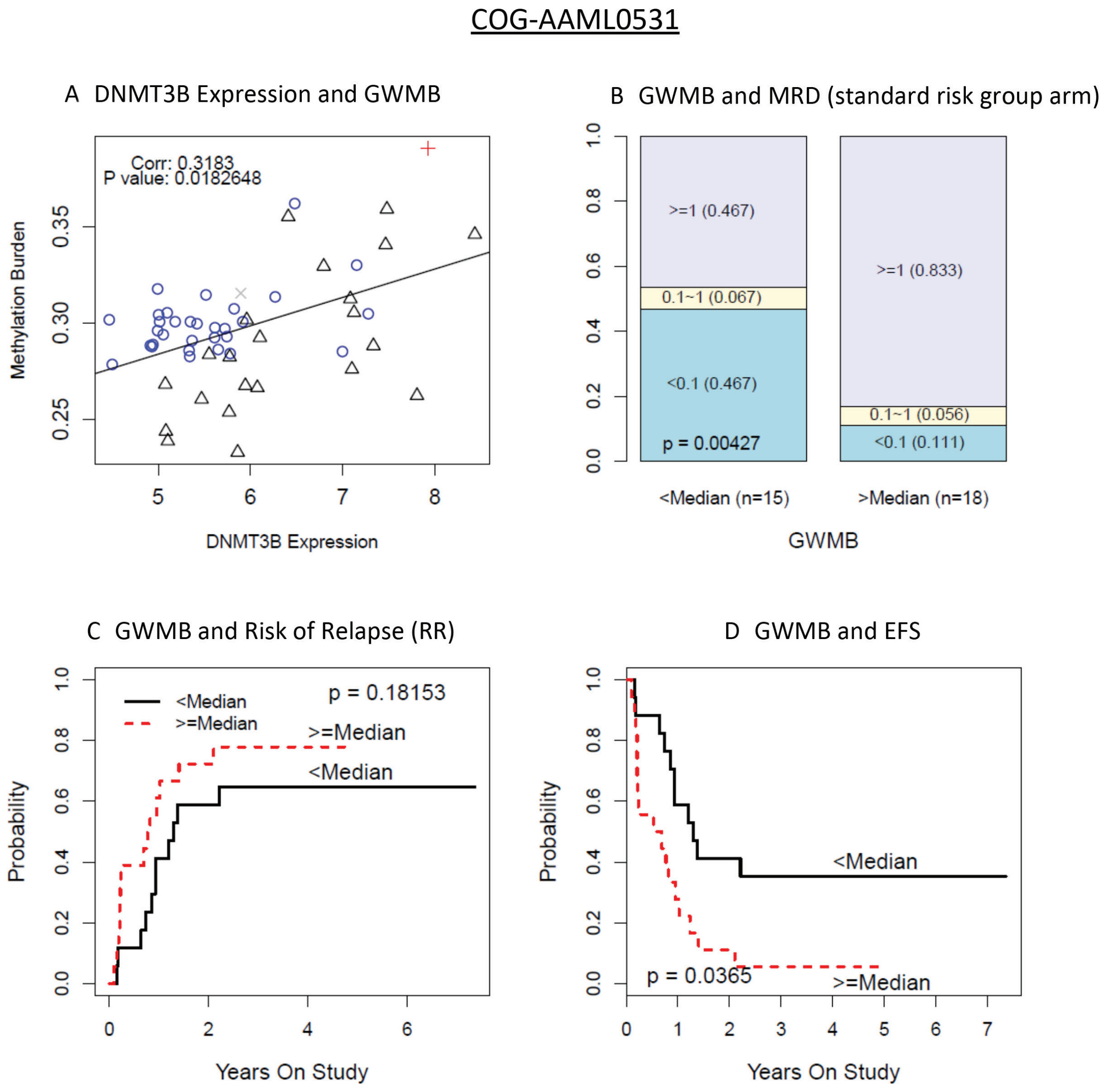

Supplementary Figure 7: DNMT3B expression impacts genome wide methylation burden (GWMB) and GWMD is associated with outcome: AAML0531 cohort. (**A**) Scatterplot showing association of 27 K array GWMB with DNMT3B expression in AAML0531. (circle: low risk; triangle: standard risk; cross: high risk). (**B**) Colored bar plot showing association of 450 K array GWMB with MRD in AAML0531 intermediate risk patients (**C** Estimates of the cumulative incidence of RR for AAML0531 intermediate risk patients by 450 K array GWMB. (**D**) Kaplan-Meier estimates of event-free survival by 450 K array GWMB.

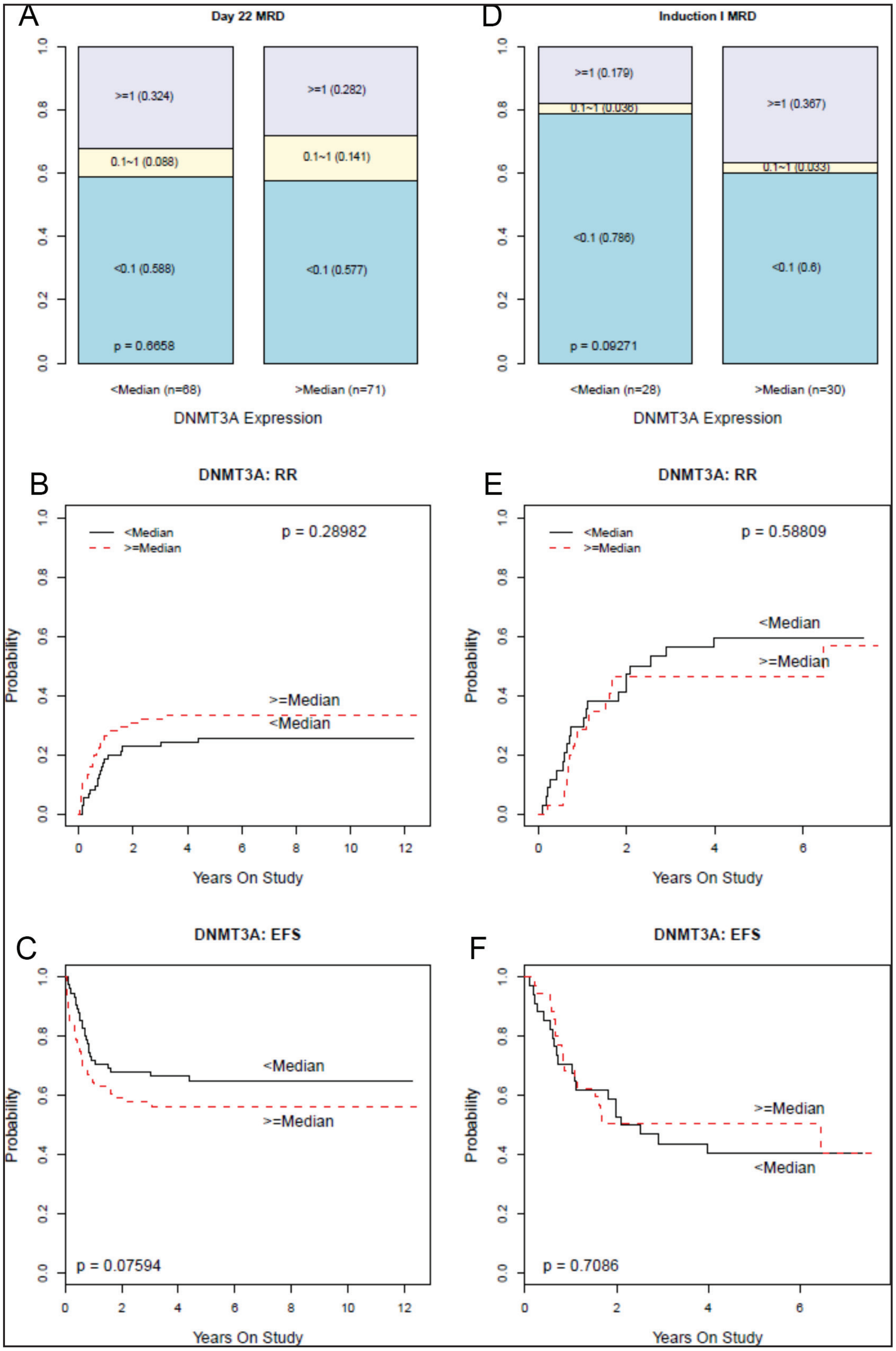

Supplementary Figure 8: DNMT3A does not show a consistent pattern of significant associations with MRD, RR, and EFS. Panels (**A**–**C**) respectively show the association of DNMT3A expression with (A) MRD, (B) RR, and (C) EFS in the AML02 cohort. Panels (**D**), (**E**), and (**F**) show analogous results for the AAML0531 cohort.

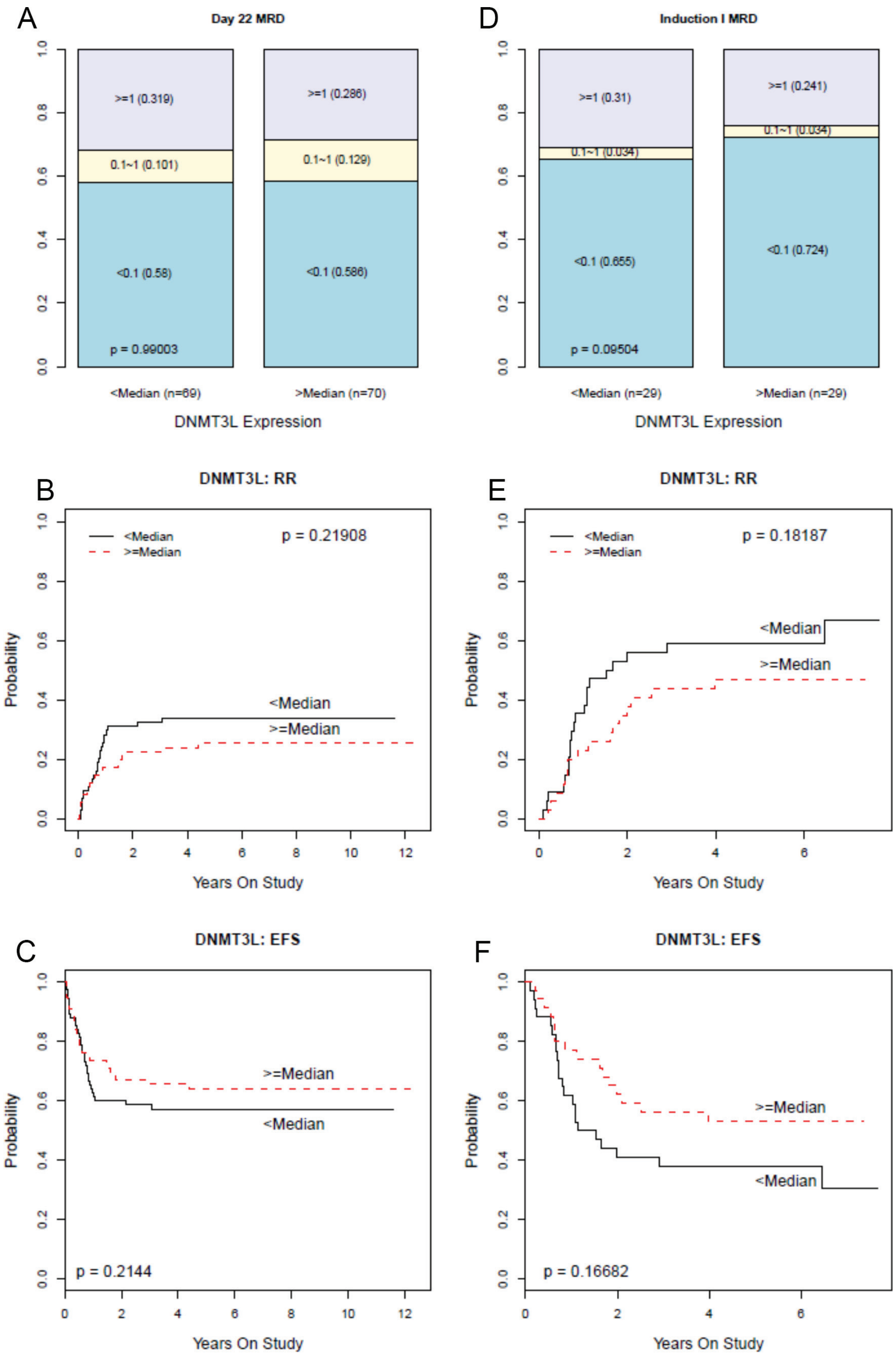

Supplementary Figure 9: DNMT3L does not show a consistent pattern of significant associations with MRD, RR, and EFS. Panels (**A–C**) respectively show the association of DNMT3L expression with (A) MRD, (B) RR, and (C) EFS in the AML02 cohort. Panels (**D**), (**E**), and (**F**) show analogous results for the AAML0531 cohort.

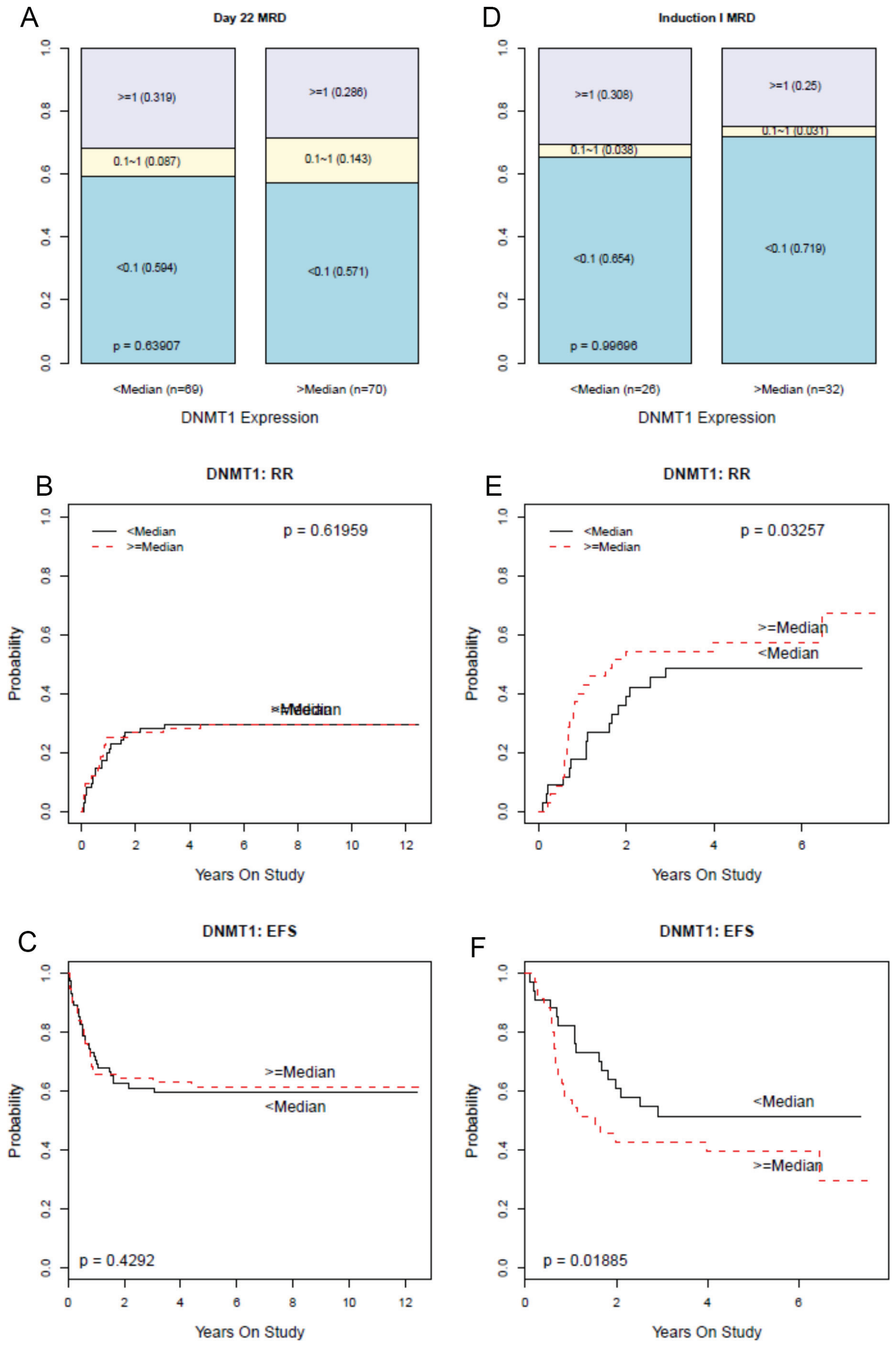

Supplementary Figure 10: DNMT1 does not show a consistent pattern of significant associations with MRD, RR, and EFS. Panels (**A**–**C**) respectively show the association of DNMT1 expression with (A) MRD, (B) RR, and (C) EFS in the AML02 cohort. Panels (**D**), (**E**), and (**F**) show analogous results for the AAML0531 cohort.
